# Comprehensive Analysis of the Prognostic Signature of Mutation-Derived Genome Instability-Related lncRNAs for Patients With Endometrial Cancer

**DOI:** 10.3389/fcell.2022.753957

**Published:** 2022-04-01

**Authors:** Jinhui Liu, Guoliang Cui, Jun Ye, Yutong Wang, Can Wang, Jianling Bai

**Affiliations:** ^1^ Department of Gynecology, The First Affiliated Hospital of Nanjing Medical University, Nanjing, China; ^2^ Department of Gastroenterology, The Second Affiliated Hospital of Nanjing University of Chinese Medicine, Nanjing, China; ^3^ The First Clinical Medical College of Nanjing Medical University, Nanjing, China; ^4^ The First Clinical Medical College, Nanjing University of Chinese Medicine, Nanjing, China; ^5^ Department of Biostatistics, School of Public Heath, Nanjing Medical University, Nanjing, China

**Keywords:** endometrial cancer, genome instability, long non-coding RNAs, risk score, prognosis predicting, immune status

## Abstract

**Background:** Emerging evidence shows that genome instability-related long non-coding RNAs (lncRNAs) contribute to tumor–cell proliferation, differentiation, and metastasis. However, the biological functions and molecular mechanisms of genome instability-related lncRNAs in endometrial cancer (EC) are underexplored.

**Methods:** EC RNA sequencing and corresponding clinical data obtained from The Cancer Genome Atlas (TCGA) database were used to screen prognostic lncRNAs associated with genomic instability *via* univariate and multivariate Cox regression analysis. The genomic instability-related lncRNA signature (GILncSig) was developed to assess the prognostic risk of high- and low-risk groups. The prediction performance was analyzed using receiver operating characteristic (ROC) curves. The immune status and mutational loading of different risk groups were compared. The Genomics of Drug Sensitivity in Cancer (GDSC) and the CellMiner database were used to elucidate the relationship between the correlation of prognostic lncRNAs and drug sensitivity. Finally, we used quantitative real-time PCR (qRT-PCR) to detect the expression levels of genomic instability-related lncRNAs in clinical samples.

**Results:** GILncSig was built using five lncRNAs (AC007389.3, PIK3CD-AS2, LINC01224, AC129507.4, and GLIS3-AS1) associated with genomic instability, and their expression levels were verified using qRT-PCR. Further analysis revealed that risk score was negatively correlated with prognosis, and the ROC curve demonstrated the higher accuracy of GILncSig. Patients with a lower risk score had higher immune cell infiltration, a higher immune score, lower tumor purity, higher immunophenoscores (IPSs), lower mismatch repair protein expression, higher microsatellite instability (MSI), and a higher tumor mutation burden (TMB). Furthermore, the level of expression of prognostic lncRNAs was significantly related to the sensitivity of cancer cells to anti-tumor drugs.

**Conclusion:** A novel signature composed of five prognostic lncRNAs associated with genome instability can be used to predict prognosis, influence immune status, and chemotherapeutic drug sensitivity in EC.

## Introduction

According to the most recent cancer statistics, endometrial cancer (EC) is the most commonly diagnosed gynecologic cancer in the United States, with estimated 65,620 new cases and 12,590 deaths (Siegel et al., 2020). However, no significant improvement in the 5-year relative survival of these patients has been achieved. The Surveillance, Epidemiology, and End Results (SEER) database contained 83.05% of data in 2018 and 82.36% in 1988 (Doll et al., 2018). EC is broadly classified into two types based on distinct pathological and clinical outcomes. Changes in multiple pathways, most notably the PTEN/PI3K/AKT/mTOR pathway, are implicated in type I EC. Moreover, the RAS/RAF/mitogen-activated extracellular signal-regulated kinase 1 (MEK)/extracellular signal-related kinase (ERK) and WNT/β-catenin signaling pathway are frequently aberrantly activated (Dong et al., 2013; Morice et al., 2016). Meanwhile, obesity and high circulating estrogen levels are strongly linked to type I EC. Other risk factors for type I EC include tamoxifen use after breast cancer, physical inactivity, hyperinsulinemia, reproductive history, and oral contraceptives (Bergman et al., 2000; Parslov et al., 2000; Kaaks et al., 2002). Type II EC has lower morbidity but a higher mortality rate than type I EC. However, this classification of EC has flaws due to the overlapping morphological and immunohistochemical characteristics of type I and II EC (Soslow, 2013).

Long non-coding RNAs (lncRNAs) are a type of transcripts, typically longer than 200 nucleotides (nt), and have no protein-coding capacity but can regulate gene expression (Evans et al., 2016). The abnormal expression of lncRNAs influences cell proliferation, tumor metastasis, and progression by interacting with specific signaling proteins, mRNAs, and micro-RNAs, allowing the formation of sophisticated networks that can contribute to phenotypic diversity in tumor cells (Dong et al., 2019). Several abnormally expressed lncRNAs have been discovered in different cancers, but their function is unknown. Tong et al. found that the lncRNA NORAD holds great promise as a prognostic biomarker in EC because it plays an important role in EC progression as a tumor suppressor (Han et al., 2020).

Genomic instability, characterized by an increased tendency for genomic changes ranging from base pair mutations to chromosomal aberrations, contributes to somatic heterogeneity and genetic diversity, as well as the progression of genetically related diseases, including cancer (Andor et al., 2017). Genomic instability is widely regarded as one of the hallmarks of cancer as it accelerates tumor progression and decreases patient survival (Negrini et al., 2010). Many studies on the classification of endometrial carcinoma have been conducted to judge the prognosis and guide treatment, and the findings revealed that the majority of endometrial carcinoma were microsatellite instable hypermutated (MSI-H) (Kandoth et al., 2013). Hypermethylation of the MMR gene promoter in MSI-H type EC resulted in the accumulation of DNA replication errors in tumor proliferation, characterized by insertion and deletion of microsatellite sequences, as well as a significantly higher genomic mutation frequency than in microsatellite-stable tumors (Zhao et al., 2014). Therefore, the degree of EC genomic instability has important diagnostic, treatment, and prognosis implications (Khanduja et al., 2016). Furthermore, because effective EC biomarkers for assessing prognosis and determining appropriate treatment are still limited, it is critical to identify novel available markers (Salvesen et al., 2012). Mounting evidence demonstrates that lncRNA is strongly linked to genomic instability (Liu, 2016). Recently, Zhaohua et al. showed that nuclear lncRNA BGL3 directly plays a role in the regulation of the DNA damage response pathway by preventing the BRCA1/BARD1 complex from accumulating on the DNA double-strand breaks (Hu et al., 2020). So far, however, few studies have been reported on this phenomenon in EC.

In this study, we attempted to integrate the expression profiles and somatic mutation profiles of patients with EC to construct a genome instability-associated lncRNAs based on risk score for prognosis prediction, immune status determination, and therapeutic scheme selection.

## Materials and Methods

### Data Acquisition and Preprocessing

The Cancer Genome Atlas (TCGA), a public database interacting with 33 cancer types, was used to obtain data on the expression profile, somatic mutation, and clinical information of endometrial carcinoma patients. The project was TCGA-UCEC, and the transcriptome data files were labeled “FPKM.” LncRNA and mRNA were extracted from the transcriptional profiling data, separately. We obtained complete lncRNA expression profiles, mRNA expression profiles, clinical features, and somatic mutation profiles for 511 samples.

### Differential lncRNA Expression and Co-Expression Analysis

The computational framework was designed to identify the lncRNAs associated with genomic instability, and then, the number of mutations was counted in each sample (Bao et al., 2020). Subsequently, we arranged subjects in descending order based on the number of somatic mutations. The patients were divided into high (≥25% mutation number) and low mutation number (<25% mutation number) groups, respectively, and designated as the genomic unstable (GU) groups and the genomic stable (GS) groups according to the mutation number. The “limma” package in R software was used to identify differentially expressed lncRNAs (DELs) between the high and low mutation number groups with a |logFC| >1 threshold and a false discovery rate adjusted *p*-value < 0.05. The co-expression network of the DELs and genes was established with a coefficient |R^2^| > 0.3 and *p* < 0.001. Next, 511 EC samples were randomly divided into training (256 patients) and testing sets (255 patients) using the R software package “caret.” Table 1 shows the clinical and pathological characteristics of the tumor samples between two sets (*p* > 0.05, Chi-square test). The preprocessing processes were as follows: (1) RNA sequencing annotation with reference file; (2) eliminating missing values; and (3) normalizing expression values by R software (3.4.0) and correcting background using the robust multichip averaging algorithm.

**TABLE 1:** Clinical features of included patients.

Covariates	Type	Total	Train	Test	*p* value
Age	<=60	199(38.94%)	93(36.33%)	106(41.57%)	0.26
	>60	312(61.06%)	163(63.67%)	149(58.43%)	
Histological_type	Endometrial	384(75.15%)	191(74.61%)	193(75.69%)	0.86
	Mixed and serous	127(24.85%)	65(25.39%)	62(24.31%)	
Grade	G1 & G2	91(17.81%)	50(19.53%)	41(16.08%)	0.37
	G3 & G4	420(82.19%)	206(80.47%)	214(83.92%)	
Stage	Stage I & Stage II	370(72.41%)	185(72.27%)	185(72.55%)	1.00
	Stage III Stage IV	141(27.59%)	71(27.73%)	70(27.45%)	

### Functional Enrichment Analysis

Gene Ontology (GO) and Kyoto Encyclopedia of Genes and Genomes (KEGG) enrichment analyses were conducted *via* the Annotation, Visualization, and Integrated Discovery (DAVID) 6.8 (https://david.ncifcrf.gov) (Yu et al., 2012). *p* < 0.05 was regarded as significantly enriched.

### Construction of Prognosis Predictive-Model and Nomogram

All patients were randomly assigned to the training and validation cohorts on a 1:1 ratio. The expression profile of DELs was then combined with prognosis data. Employing the survival package, the prognosis prediction model was developed with the formula of “Riskscore = ∑coef∗Exp(genes) ”. Patients with risk scores higher than the median were assigned to the high-risk group, whereas others were assigned to the low-risk group. The Kaplan–Meier survival curve was used to compare the prognosis difference of high- and low-risk groups (Liu et al., 2020a). The receiver operating characteristic (ROC) curve was used to assess the predictive potential of our prognosis model. Dimensionality was reduced using principal component analysis (PCA) (Liu et al., 2019). Risk scores and clinical characteristics of patients were combined to establish a nomogram for improved predictive ability, which was then tested using calibration plots. Genomic instability-related lncRNA signature (GILncSig) prognosis was assessed using univariate and multivariate Cox regression analyses, as well as stratified analyses. We compared the areas under the ROC curves (AUCs) to evaluate GILncSig performance.

### Immune Microenvironment, Checkpoint Analysis, and Gene Set Enrichment Analysis

The immune cell landscape in the tumor microenvironment was quantified by ssGSEA algorithms (Zhu et al., 2016). When compared to traditional quantification algorithms, ssGSEA had a higher degree of freedom in calculating multiple immune cell scores based on the expression profile. The expression of immune checkpoint genes was also examined in the high- and low-risk groups to investigate the underlying effect on immunotherapy. GSEA (http://software.broadinstitute.org/gsea/index.jsp) was used to identify biological processes with a high gene rank. The EC samples in the TCGA set were divided into high-risk and low-risk groups based on the riskscore model. The underlying biological functions of the two groups were identified by comparing the enrichment of biological processes. The collection of annotated gene sets in the Molecular Signatures Database (MSigDB, http://software.broadinstitute.org/gsea/msigdb/index.jsp) served as the reference gene set in GSEA software. The Nom. *p <* 0.05 was chosen as the cutoff criterion (Song et al., 2017). The c2.cp.kegg.v7.4.symbols.gmt was chosen as the reference file.

### Immunophenoscore Analysis

The immunophenoscores (IPSs) of patients with EC were obtained from The Cancer Immunome Atlas (https://tcia.at/home). The IPS analysis was performed randomly using machine learning to determine the immunogenicity of four categories of genes: effector cells, suppressive cells, major histocompatibility complex molecules, and immune modulators or checkpoints. The IPS was calculated using weighted averaged Z-scores from the above categories with a range of 0–10, and the scores were increased with higher immunogenicity (Charoentong et al., 2017).

### ESTIMATE Algorithm

Estimation of Stromal and Immune cells in Malignant Tumours using Expression data (ESTIMATE) is an algorithm for determining the levels of stromal and immune cell infiltration in tumors (Yoshihara et al., 2013). ESTIMATE algorithm was used to evaluate the immune cell content (immune score), stromal cell infiltration (stromal score), strom-immune synthesis score (ESTIMATE score), and tumor purity for each EC sample.

### TMB Calculation and Clinical Data Analysis

The tumor mutation burden (TMB) level was calculated by dividing the total number of mutations by the size of the coding region of the target region. The EC samples were divided into high-TMB and low-TMB groups based on the median TMB level. The TMB level from the TCGA database was combined with the corresponding survival data from each sample, and the differences in OS between different TMB level groups and different TMB levels combined with different risk groups were compared using Kaplan–Meier analysis. The Wilcoxon rank sum test was used to investigate the correlation of TMB with the risk score.

### Somatic Mutation, Tumor Stemness, and Drug Sensitivity Analysis

The mutation data of endometrial carcinoma patients were obtained from the TCGA (Data Category = copy number variation; “maf” file). Fall plots were used to visualize the top 10 mutation genes *via* the maftools packages in R software (Mayakonda et al., 2018). As previously reported, the stem-like indices for each endometrial carcinoma sample were calculated using one-class logistic regression (Yi et al., 2020). Then, in the high-risk and low-risk groups of GILncSig, we estimated the half-maximal inhibitory concentration (IC50) of selected drugs from a public database called Genomics of Drug Sensitivity in Cancer (GDSC; https://www.cancerrxgene.org) using the “pRRophetic” packages (Song et al., 2020). The NCI-60 database, which was assessed *via* the CellMiner interface (https://discover.nci.nih.gov/cellminer), is currently the most widely used for cancer drug testing (Dong et al., 2020). Pearson correlation analysis was performed to explore the underlying drug sensitivity difference between the high- and low-risk groups.

### Quantitative Real-Time PCR

A total of 15 paired EC tissues and normal tissues were obtained from patients at the First Affiliated Hospital of Nanjing Medical University. The Ethics Committee of the First Affiliated Hospital of Nanjing Medical University (Nanjing, China) approved this study. All patients signed informed consent forms. Total RNA was isolated from samples using TRIZOL reagent (Thermo Fisher Scientific, USA), then reverse-transcribed into cDNA using a Revert Aid First Strand cDNA Synthesis kit (Thermo Fisher Scientific, USA), and analyzed by quantitative real-time PCR (qRT-PCR) with an SYBR-Green PCR kit (Takara, Tokyo, Japan). GAPDH was used to normalize the relative expression of the lncRNA. The sequences are listed in Supplementary Table S1.

### Statistical Analysis

All the analyses were performed using the R software (version 4.1.0). All statistical tests were two-sided, with a *p*-value of less than 0.05 considered statistically significant. The Student t-test was used to compare the normally distributed variables in two groups, and the Wilcox test was used to compare non-normally distributed continuous variables.

## Results

### Sample Clustering and Enrichment Analysis

**FIGURE 1:**
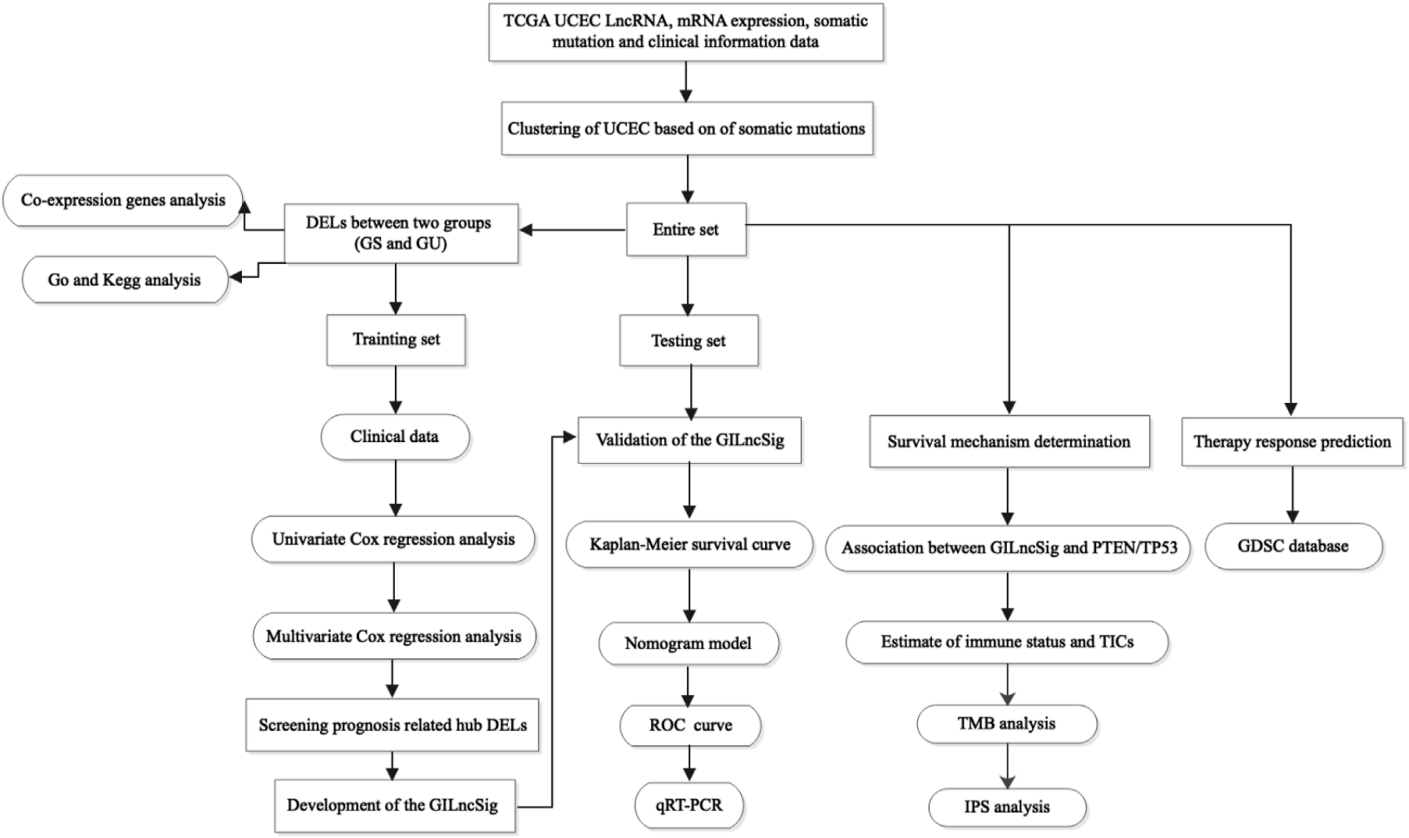
Research roadmap of this study.

**FIGURE 2:**
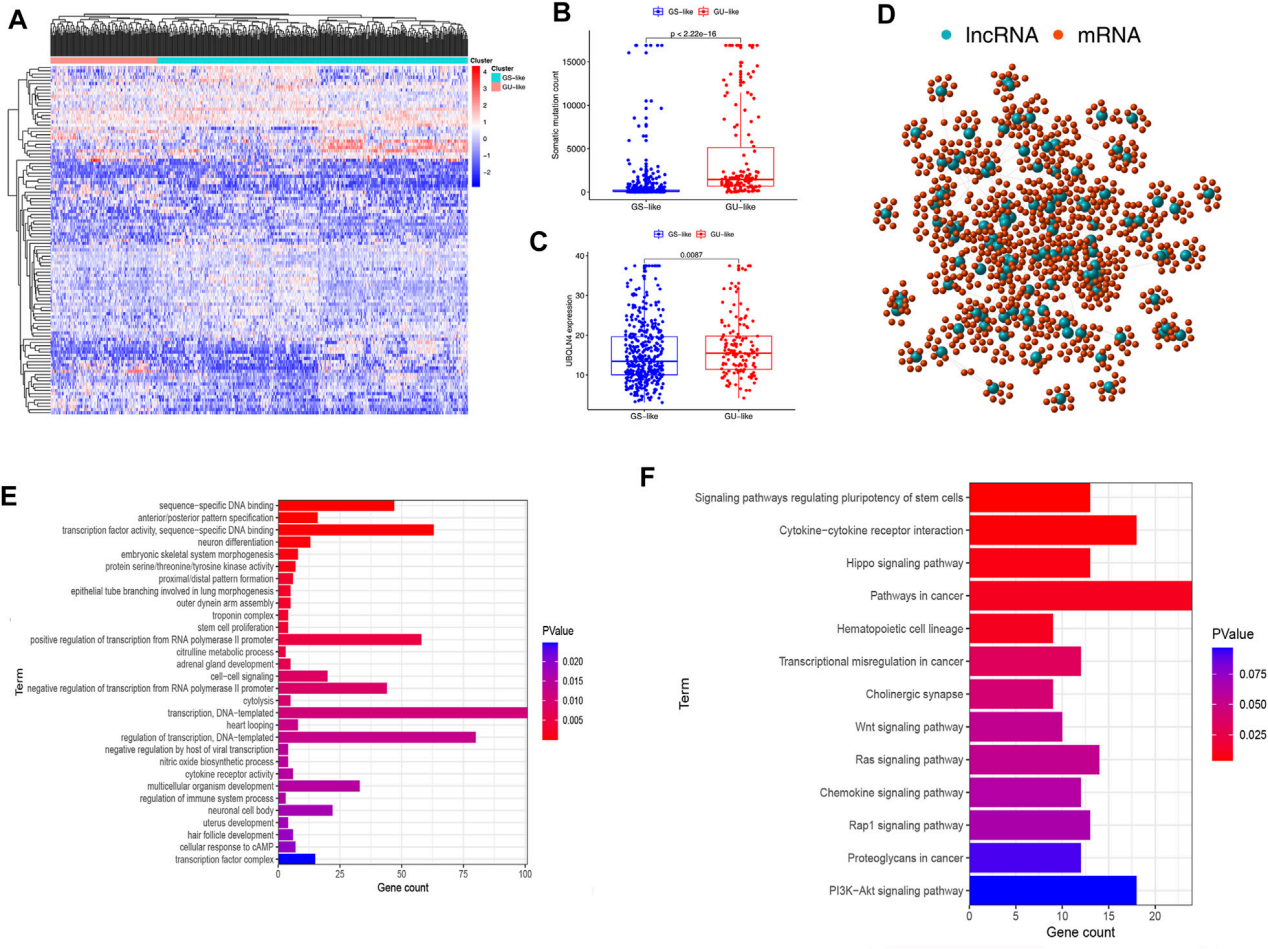
Cluster analysis and functional annotation of genomic instability-related lncRNAs in patients with endometrial carcinoma. **(A)** Unsupervised clustering was performed on patients with endometrial carcinoma based on the expression patterns of 128 candidate genomic instability-related lncRNAs. **(B)** Level of somatic mutation count in GU and GS groups. **(C)** Expression level of UBQLN4 in GU and GS groups. **(D)** Co-expression network of genomic instability-related lncRNAs and mRNAs based on Pearson correlation coefficient. **(E,F)** Results of Gene Ontology analysis and Kyoto Encyclopedia of Gene and Genomes pathway enrichment analysis for lncRNAs co-expressed mRNAs.

Figure 1 depicts the research procedure for this study. According to the cumulative somatic mutations, we assigned the top 25% highest mutation and the bottom 25% lowest mutation to the GU (n = 133) and GS groups (n = 131). There were 109 DELs between the two groups, with 31 lncRNAs upregulated and 78 lncRNAs downregulated in the GU group (adj. *p*-value < 0.05, |logFC| > 1) (Supplementary Table S2). Supplementary Figure S1 shows a heatmap of 20 most significantly upregulated lncRNAs and 20 most significantly downregulated lncRNAs. Unsupervised hierarchical clustering analysis was used to classify all TCGA samples into GU (higher cumulative somatic mutations) and GS (lower cumulative somatic mutations) groups based on the set of 109 DELs (Figure 2A). As one of the members of the ubiquitin-like and ubiquitin-associated (UBL-UBA) protein family, UBQLN4 plays an important role in sustaining genomic stability by affecting nucleotide excision repair. Recent studies have revealed that the mutation of UBQLN4 could reflect genomic instability in cancers, thus affecting prognosis and the chemotherapy response (Jachimowicz et al., 2019). Therefore, UBQLN4 was selected to reflect the genomic instability. Similarly, the GU-like group had a higher level of somatic mutation count and UBQLN4 expression (Figures 2B,C). Meanwhile, a co-expression network of these lncRNAs and mRNA was constructed (Figure 2D). GO analysis revealed that the lncRNAs were significantly enriched in “sequence-specific DNA binding,” “anterior/posterior pattern specification,” “transcription factor activity, sequence-specific DNA binding”, “neuron differentiation”, and “regulation of immune system process” (Figure 2E). KEGG analysis demonstrated that the lncRNAs were significantly enriched in “signaling pathways regulating pluripotency of stem cells,” “cytokine-cytokine receptor interaction”, “hippo signaling pathway,” “pathways in cancer”, and “hematopoietic cell lineage” (Figure 2F). As of now, 109 lncRNAs have been identified as genome instability-related lncRNAs.

### Constructing the OS Prediction Model

**FIGURE 3:**
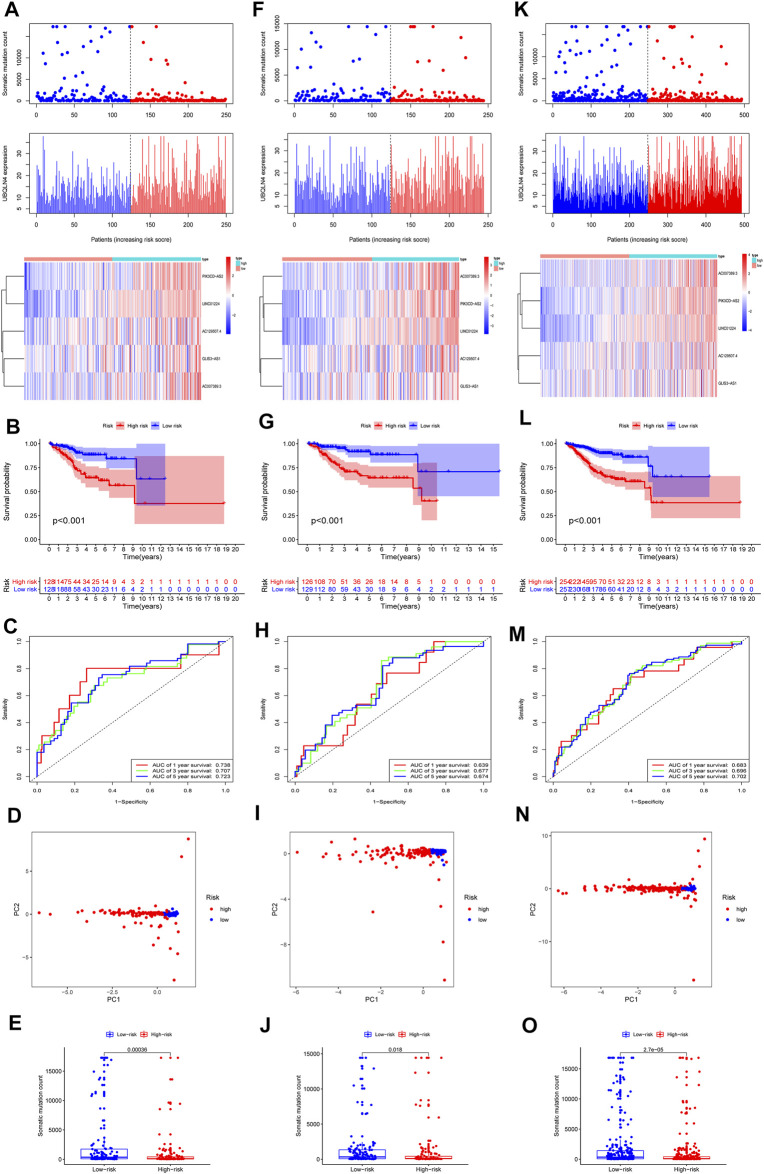
Construction of the GILncSig for prognostic prediction model of genomic instability-related lncRNA. In training set, **(A)** distribution of somatic mutation, the expression level of UBQLN4 and five lncRNAs expression patterns as the risk score increased. **(B)** Prognostic prediction in the high-risk group and low-risk group by Kaplan–Meier. **(C)** Time-dependent ROC curves were used to verify the survival prediction model. **(D)** PCA analysis revealed the difference between the high- and low-risk groups. **(E)** Level of somatic mutation count in the high- and low-risk groups. GILncSig evaluation in the testing set. **(F)** Distribution of somatic mutation and expression level of UBQLN4 as well as expression patterns of five lncRNAs as the risk score increased. **(G)** Prognostic prediction in the high- group and low-risk groups by Kaplan–Meier. **(H)** Time-dependent ROC curves were used to verify the survival prediction model. **(I)** PCA analysis revealed the difference between the high- and low-risk groups. **(J)** Level of somatic mutation count in the high- and low-risk groups. GILncSig evaluation in the TCGA set. **(K)** Distribution of somatic mutation and expression levels of UBQLN4 as well as expression patterns of five lncRNAs as the risk score increased. **(L)** Prognostic prediction in the high- and low-risk groups by Kaplan–Meier. **(M)** Time-dependent ROC curves were used to verify the survival prediction model. **(N)** PCA analysis revealed the difference between the high- and low-risk groups. **(O)** Level of somatic mutationcount in the high-risk and low-risk groups. Red color represents the high-risk group, and blue color represents the low risk-group.

We randomly divided 511 EC samples into a training group (*n* = 256) and a test group (*n* = 255) to investigate the prognostic value of 109 candidate lncRNAs associated with genomic instability. The Chi-square test and the Wilcoxon rank sum test were used to demonstrate that no significant differences in clinicopathological covariates existed between the training and the testing groups (Table 1). We used univariate and multivariate Cox proportional hazards regression to select five of 14 candidate lncRNAs as prognostic lncRNAs (Supplementary Tables S3, S4). Finally, in the training cohort, the lncRNAs AC007389.3, PIK3CD-AS2, LINC01224, AC129507.4, and GLIS3-AS1 were included in our prognosis model with the formula of “Riskscore = AC129507.4 *0.0336 + GLIS3-AS1 * 0.0183 + PIK3CD-AS2 *0.1192 + LINC01224 *0.1728 + AC007389.3 *0.2166”. Then, on the basis of the median value of the risk score, we calculated the risk score for each EC sample in the training set and divided it into the low-risk group (*n* = 128) and high-risk group (*n* = 128). The patients with EC were ranked in ascending order based on the risk score and observed the changes in the trend of GILncSig, the number of somatic mutations, and the level of expression of UBQLN4 (Figure 3A). The heatmap compared clinicopathological features between two groups based on the expression of five lncRNAs associated with genomic instability. The analysis revealed significant differences in terms of stage, grade, histological, type, and age (Supplementary Figure S2, p < 0.01). In the Kaplan–Meier analysis, we found that overall survival (OS) was higher in the low-risk group than that in the high-risk group (Figure 3B). According to the ROC curve, our GILncSig model exhibited good sensitivity and specificity in predicting the OS of patients with EC (5 years, AUC = 0.723; 3 years, AUC = 0.707; 1 year, AUC = 0.738) (Figure 3C). The PCA revealed that samples from the two groups were distributed in opposite directions (Figure 3D). Meanwhile, it was discovered that the number of somatic mutations was lower in the high-risk group than that in the low-risk group (Figure 3E).

### Validating the Prognostic Prediction Model in the Testing Set

Using the same risk scoring formula as in the training set, a total of 255 samples in the testing set were divided into the low-risk group (*n* = 129) and high-risk group (*n* = 126), and the predictive power was demonstrated in the testing set. The changes in the GILncSig trend, number of somatic mutations, and UBQLN4 expression level in the training set were similar to those in the testing set (Figure 3F). Moreover, the testing set exhibited prognostic patterns similar to the testing set (Figure 3G). The ROC curve demonstrated that the model had good sensitivity and specificity in predicting patient OS in the testing set (5 years, AUC = 0.674; 3 years, AUC = 0.677; 1 year, AUC = 0.639) (Figure 3H). PCA analysis displayed a different distribution pattern for the low-risk and high-risk groups according to the expression of five lncRNAs in the testing set (Figure 3I). The number of somatic mutations in the high-risk group was lower than in the low-risk group (Figure 3J).

### Validating the Prognostic Prediction Model in the TCGA Set

The 511 samples To in the TCGA set were stratified into low-risk (*n* = 257) and high-risk groups (*n* = 254) based on the cutoffs in the training set to further validate the prediction model. The distribution patterns of GILncSig, somatic mutation number, and UBQLN4 expression level were consistent with the above results for training and testing sets (Figure 3K). In addition, the high-risk group had a much lower OS than the low-risk group (Figure 3L). The ROC curve revealed that the GILncSig in the TCGA set had good sensitivity and specificity in predicting patient OS in the testing set (5 years, AUC = 0.702; 3 years, AUC = 0.696; 1 year, AUC = 0.683) (Figure 3M). In the TCGA set, PCA analysis displayed a distinct distribution pattern for the low-risk and high-risk groups (Figure 3N). The number of somatic mutations in the high-risk group was lower than in the low-risk group (Figure 3O). Furthermore, GSEA was used to examine the transcript message of genomic instability-related lncRNAs in the high-risk and low-risk subgroups. Representative KEGG pathways in the high-risk subgroup model were “base excision repair,” “cell cycle,” “DNA replication,” “mismatch repair,” (MMR) and “nucleotide excision repair.” Representative KEGG pathways in the low-risk subgroup model were “asthma,” “autoimmune thyroid disease,” “cytokine-cytokine receptor interaction,” “graft-versus-host disease,” and “intestinal immune network for IgA production” (Supplementary Figure S3).

### ROC Analysis of Clinical Factors and the Risk Score

**FIGURE 4:**
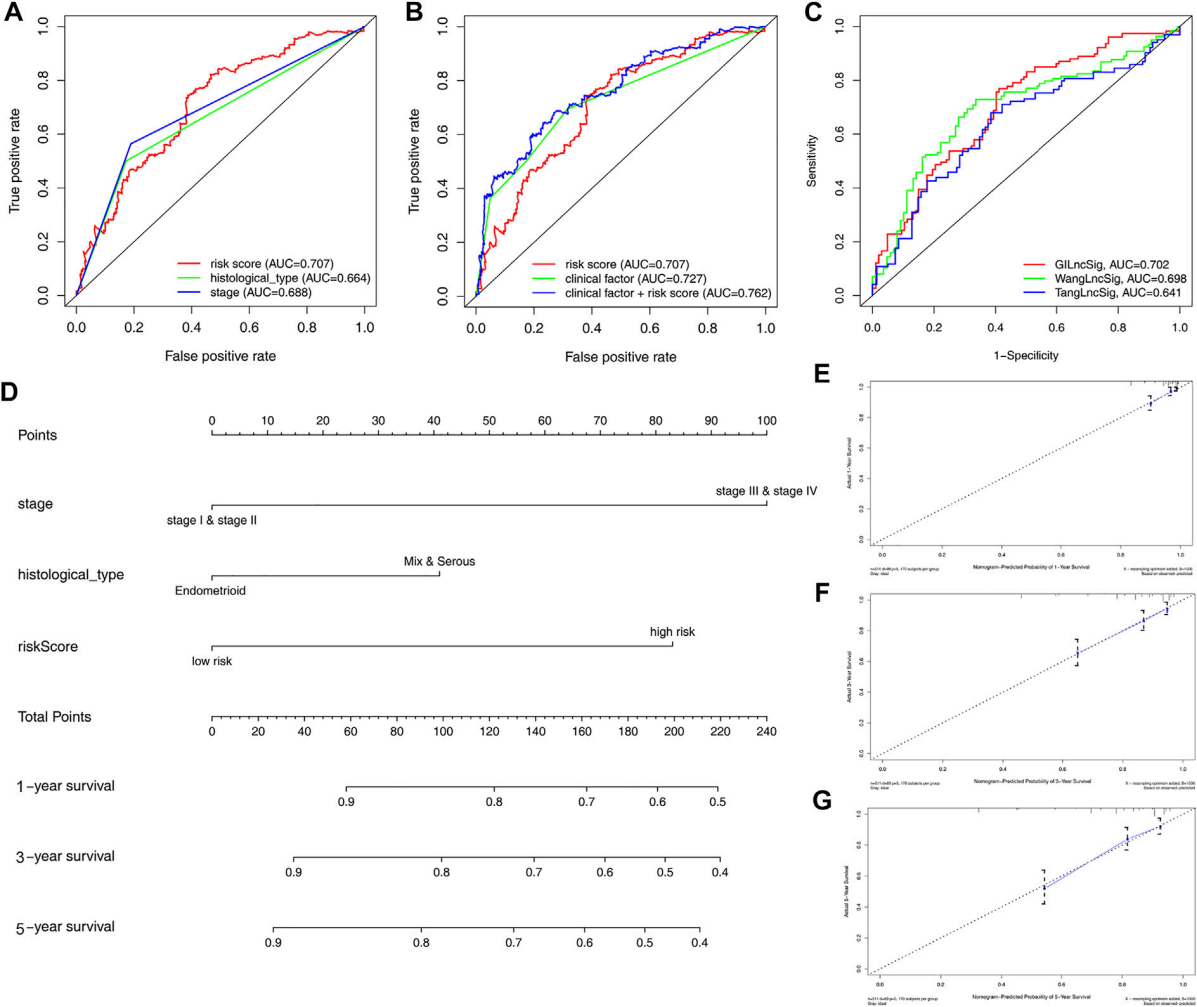
Construction of nomogram for predicting survival of patients with EC. **(A)** Time-dependent ROC analysis to evaluate prognostic value of the risk score and clinical factors (histological type and stage). **(B)** Time-dependent ROC analysis based on risk score and merged clinical factors. **(C)** Time-dependent ROC analysis of OS for the GILncSig, WangLncSig, and TangLncSig. **(D)** The baseline nomogram from three clinicopathological parameters (stage, histological type, and risk score). **(E–G)** Calibration plots of 1-, 3-, and 5-year OS for the patient with predicting.

ROC curve analysis revealed that the AUC of risk score was 0.707 and the clinical factor was 0.727 in this model, both significantly higher than histological type (AUC = 0.664) and stage (AUC = 0.688). Intriguingly, the AUC of clinical factor plus risk score (AUC = 0.762) was the highest in this ROC curve (Figures 4A,B). The ROC curve demonstrated a better predictive potential of GILncSig; however, combining clinical factors and GILncSig yielded the best predicting effect on OS in patients with EC. To demonstrate further the predictive performance of the ROC curve for lncRNA in this model, we compared two recently published articles on the signatures of lncRNAs (Tang et al., 2019; Wang et al., 2020a). Using the same TCGA patient cohort, it was revealed that the AUC of OS for the GILncSig in this model is 0.702, significantly higher than other models such as WangLncSig (AUC = 0.698) and TangLncSig (AUC = 0.641) (Figure 4C). As such, a nomogram incorporating risk score and clinical factors were developed to provide clinicians with a quantitative method for predicting OS in patients with EC (Figure 4D). The calibration curve of 1-, 3-, and 5-year survival showed that the nomogram was nearly an ideal model (Figures 4E–G). The prognostic factors of patients with EC are shown in Supplementary Figure S4; it was revealed that age, grades 3 and 4, histological type, and stage were the most important factor affecting the prognosis of patients with EC, whereas initial stage (grades 1 and 2) did not affect prognosis between the high-risk and low-risk groups. These findings suggest that, compared to nomograms based on lncRNA signatures, those based on GILncSig may better predict outcome and clinical features for patients with EC, thereby aiding clinical management.

### Assessing the Independent Prognostic Significance of Risk Score and Clinical Stratification Analysis

We performed univariate and multivariate Cox regression analyses on three datasets (training, testing, and TCGA) for variables, including age, histological type, stage, grade, and risk score to see if it is an independent prognostic factor from the clinicopathological features. After adjusting for other clinical factors, including age, gender, and tumor stage, clinical stratification analyses of the prognostic performance of GInLncSig in the TCGA dataset revealed that patients in the low-risk group had better survival outcomes than those in the high-risk group across all clinically stratified subgroups (*p* < 0.05, log-rank test; Table 2). These results suggested that the prognostic significance of GInLncSig in patients with EC is independent of other clinicopathological variables.

**TABLE 2:** Univariate and multivariable Cox regression analysis of the GILnvSig and overall survival in different patient sets.

Variable	Univariable model			Multivariable model		
	HR	95% CI	*p*-value	HR	95% CI	*p*-value
Training set (*n* = 256)						
Risk score	1.216	1.133–1.305	5.54E-08	1.176	1.087–1.272	4.97E-05
Age	1.337	0.7116–2.513	0.368			
Grade	2.965	1.062–8.282	0.038	1.566	0.514–4.771	0.430
Stage	3.154	1.765–5.636	0.000	2.220	1.170–4.212	0.015
Histological_type	2.216	1.228–3.999	0.008	1.439	0.774–2.676	0.250
Testing set (*n* = 255)						
Risk score	1.000	1.000–1.000	0.006	1.000	1.000–1.000	0.031
Age	2.447	1.204–4.973	0.013	1.713	0.810–3.619	0.159
Grade	4.170	1.007–17.266	0.049	1.684	0.379–7.475	0.493
Stage	5.806	3.036–11.104	1.06E-07	3.906	1.946–7.840	0.000
Histological_type	4.247	2.320–7.774	2.75E-06	2.477	1.253–4.894	0.009
TCGA set (*n* = 511)						
Risk score	1.000	1.000–1.000	0.003	1.000	1.000–1.000	0.020
Age	1.778	1.112–2.843	0.016	1.582	0.967–2.589	0.068
Grade	3.363	1.467–7.770	0.004	1.532	0.632–3.713	0.345
Stage	4.116	2.670–6.275	4.82E-11	3.252	2.055–5.1483	4.82E-07
Histological_type	3.044	2.003–4.624	1.84E-07	1.875	1.179–2.9	0.008

### Prediction Performance Between the GILncSig and PTEN

**FIGURE 5:**
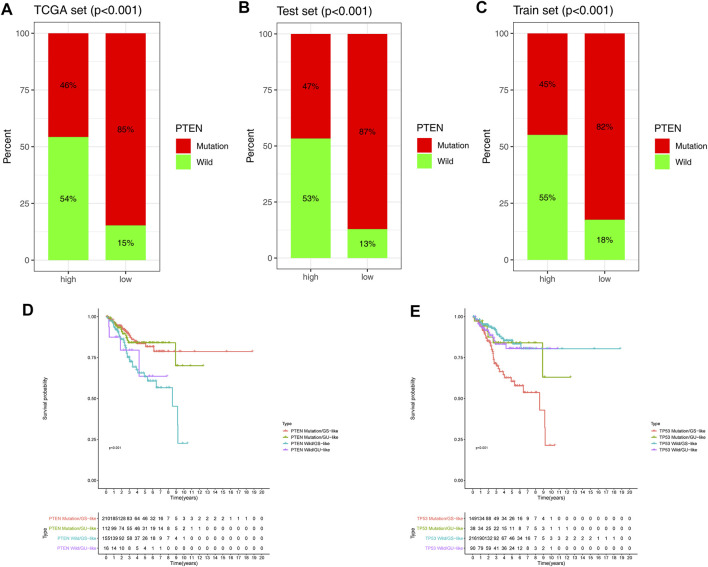
Combined survival analysis of the GILncSig and PTEN mutation. Proportion of PTEN mutation in the high- and low-risk groups in the TCGA set **(A)**, testing set **(B)**, and training set **(C)**. **(D,E)** Kaplan–Meier curves were used to analyze the OS of PTEN and TP53 in mutation/GS-like, mutation/GU, wild/GS, and wild/GU groups.

Previous research has demonstrated that the phosphate and tension homolog deleted on chromosome 10 (PTEN) gene plays a critical role in EC progression (Wang et al., 2021). To learn more about the relationship between the GILncSig and PTEN mutations, we examined the proportion of patients with PTEN mutations in three groups. The analysis showed that PTEN mutations in the low-risk group were significantly higher than those in the high-risk group in the TCGA, testing, and training sets (Figures 5A–C). However, it is unclear whether higher PTEN mutation was a better predictor of survival outcomes than GILncSig. Compelling evidence shows that the TP53 mutation is an important prognostic factor in EC (Hollis et al., 2020), as such, we compared the predictive performance of GILncSig and the TP53 mutation simultaneously. PTEN/TP53-sequence wild type (PTEN/TP53 wild) and PTEN/TP53-sequence mutation type (PTEN/TP53 mutation) EC patients were divided into GU and GS groups. PTEN wild EC patients in the GS groups (defined as PTEN wild/GS-like) had a worse prognosis than PTEN mutation EC patients in GS groups (defined as PTEN wild/GS-like) (*p* < 0.001) and PTEN mutation EC patients in GU groups (defined as PTEN mutation/GU-like) (*p* < 0.001). Whereas, TP53 mutation in the GS groups (defined as TP53 mutation/GS-like) had worse OS than TP53 wild in the GS groups (defined as TP53 wild/GS-like) (*p* < 0.001) and in the GU groups (defined as TP53 wild/GU-like) (*p* < 0.05). Meanwhile, there was no difference in survival between the other groups (Figures 5D–E). These findings demonstrate that GILncSig holds great promise as an effective tool in predicting OS as PTEN mutation and TP53 mutation.

### Analysis of the Mutation Profiles of Two Risk Groups

**FIGURE 6:**
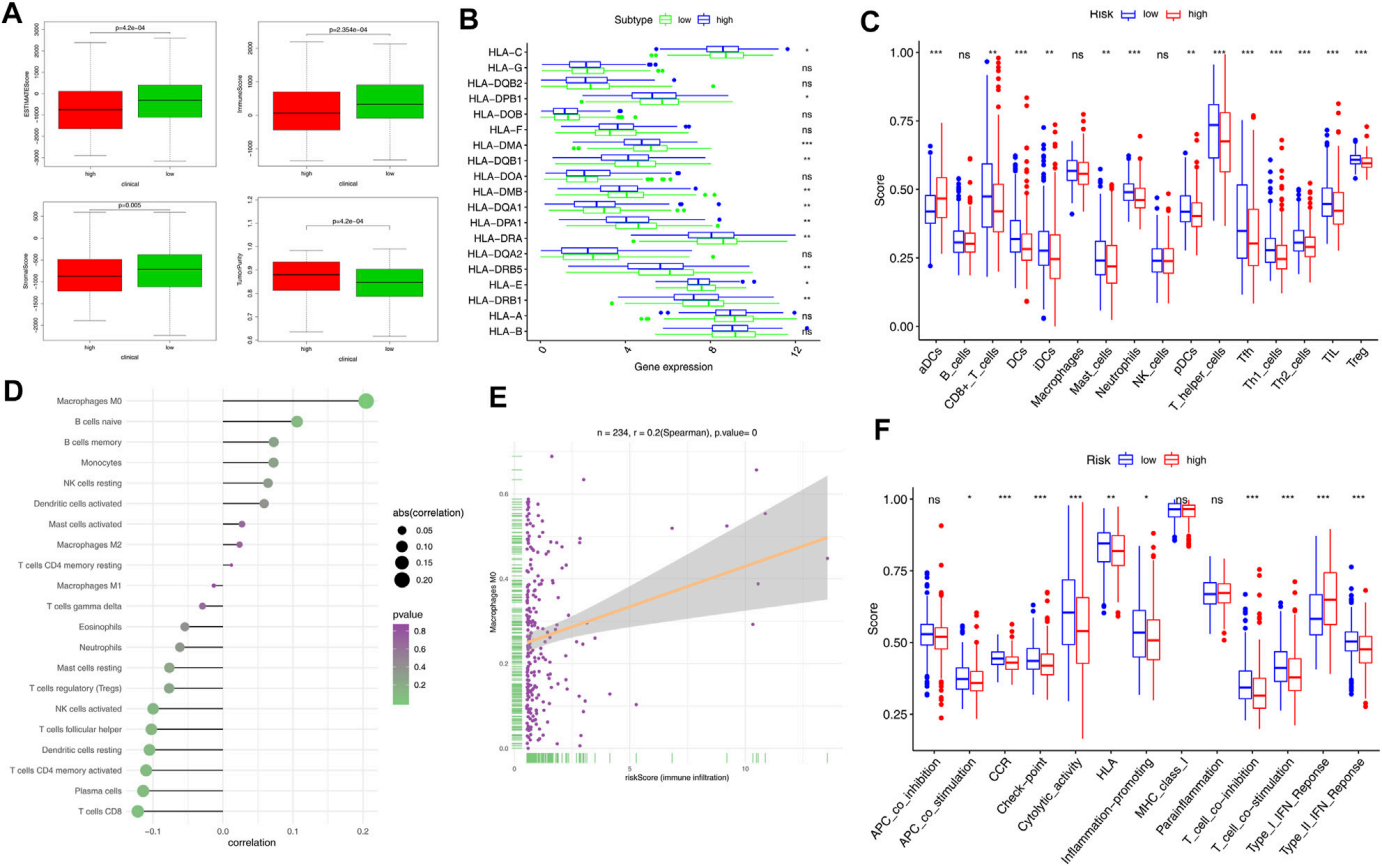
Comparison of immune signature between the high- and low-risk groups of patients with EC. **(A)** Comparison of ESTIMATES score, immune score, stromal score, and tumor purity in the high- and low-risk groups. **(B)** Expression of HLA-related genes in different risk groups. **(C)** Comparison of tumor-infiltrating immune cells (TIICs). **(D)** Correlation of risk score and TIICs. **(E)** Association of Macrophages M0 and risk score (immune infiltration). **(F)** Scores of 13 immune-related functions in the high- and low-risk groups. Adjusted *p*-values were shown as follows: ns, not significant; *p < 0.05; **p < 0.01; and ***p < 0.001.

To assess the immune status in the high-risk and low-risk groups, we compared the ESTIMATE scores (including immune scores and stromal scores) with tumor purity. The ESTIMATE algorithm results revealed that the ESTIMATE score, immune score, and stromal score of the high-risk group were lower than those of the low-risk group. Tumor purity, on the other hand, was higher in the high-risk group (Figure 6A). Human leukocyte antigen (HLA)–related genes had significant effects on immune regulation, and HLA-related gene expression was significantly lower in the high-risk group compared to the low-risk group (Figure 6B). Previous evidence shows that lncRNAs mediate the function of tumor-infiltrating immune cells (TICs) (Athie et al., 2020); therefore, we looked at the relationship between five prognostic lncRNAs and TICs (Supplementary Figure S5). Immune cells significantly impact the tumor immune microenvironment; as such, we compared tumor immune-related cells across the risk groups. The results showed that the abundances of aDCs were significantly decreased in the low-risk group, whereas the abundances of CD8^+^ T cells, DCs, iDCs, mast cells, neutrophils, pDCs, T helper cells, Tfh, Th1 cells, Th2 cells, TIL, and Treg cells were significantly decreased in the high-risk group (Figure 6C). The correlation of risk score and tumor immune-related cells, as well as the most relevant tumor immune cells, are illustrated in Figures 6D,E. Comparisons of 13 immune-related functions confirmed the difference of APC costimulation, CCR, checkpoint, cytolytic activity, HLA, inflammation-promoting, T cell co-inhibition, T cell costimulation, type I IFN response, type II IFN response in the high-risk and low-risk groups (Figure 6F).

**FIGURE 7:**
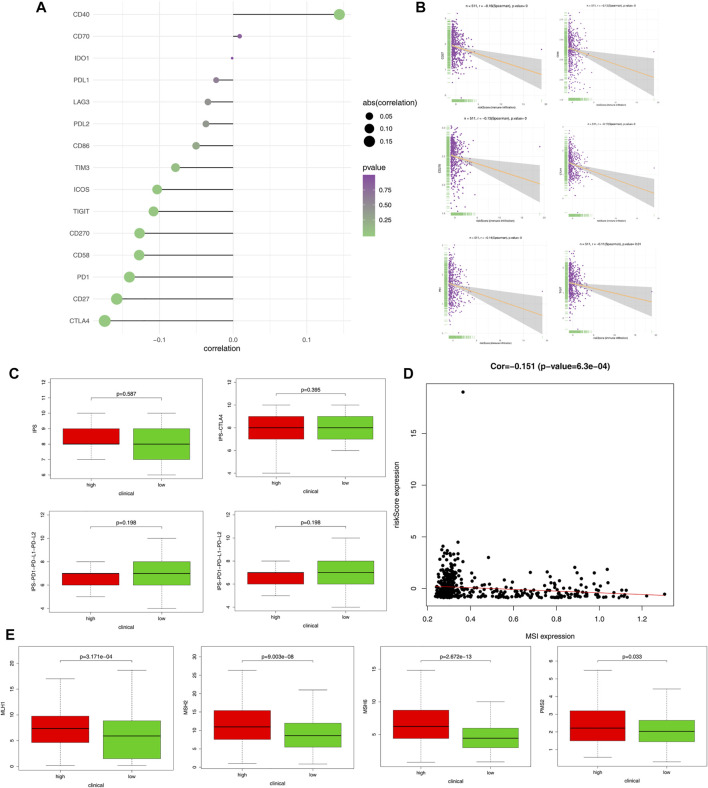
Immunophenoscore (IPS) score and gene expression analysis of immune checkpoint. **(A)** Correlation of risk score and expression of ICI related genes. **(B)** Correlation of CD27, CD58, CD270, CTLA4, PD1, TIGIT, and risk score. **(C)** Expression level of IPS, IPS-CTLA4, IPS-PD1-PD-L1-PD-L2, and IPS-PD1-PD-L1-PD-L2-CTLA4 in the high- and low-risk groups of patients with endometrial cancer. **(D)** Negative correlation between immune-related risk score and microsatellite instability (MSI). **(E)** The gene expression of MLH1, MSH2, MSH6, and PMS2 in the high- and low-risk groups.

Immune checkpoint proteins were involved in immune response and interacted with immune cells in various ways. Among many immune checkpoint proteins, CD27, CD40, CD70, CD270, B7-H3, B7-H4, IDO1, PD-1, PD-L1, PD-L2, TIM-3, TIGIT, CTLA4, CD86, ICOS, and LAG3 have been extensively studied and are thought to be important immunomodulators. First, we looked at the correlations between immune checkpoint proteins and risk score (Figure 7A); the top six immune checkpoint proteins that were most negatively associated with the risk score are shown in Figure 7B. The scores of IPS, IPS-CTLA4, IPS-PD1-PD-L1-PD-L2, and IPS-PD1-PD-L1-PD-L2-CTLA4 in the high-risk and low-risk groups were calculated to evaluate immune signature and predict the potential role of immune checkpoint inhibitors (Figure 7C). Moreover, we found that the risk score was negatively correlated with MSI (*p* = 0.00063, Figure 7D). In addition, the expression levels of MLH1, MSH2, MSH6, and PMS2 were significantly higher in the high-risk group compared to the low-risk group (Figure 7E).

### Genome-Wide Mutation Profiling in EC Between Two Risk Groups

**FIGURE 8:**
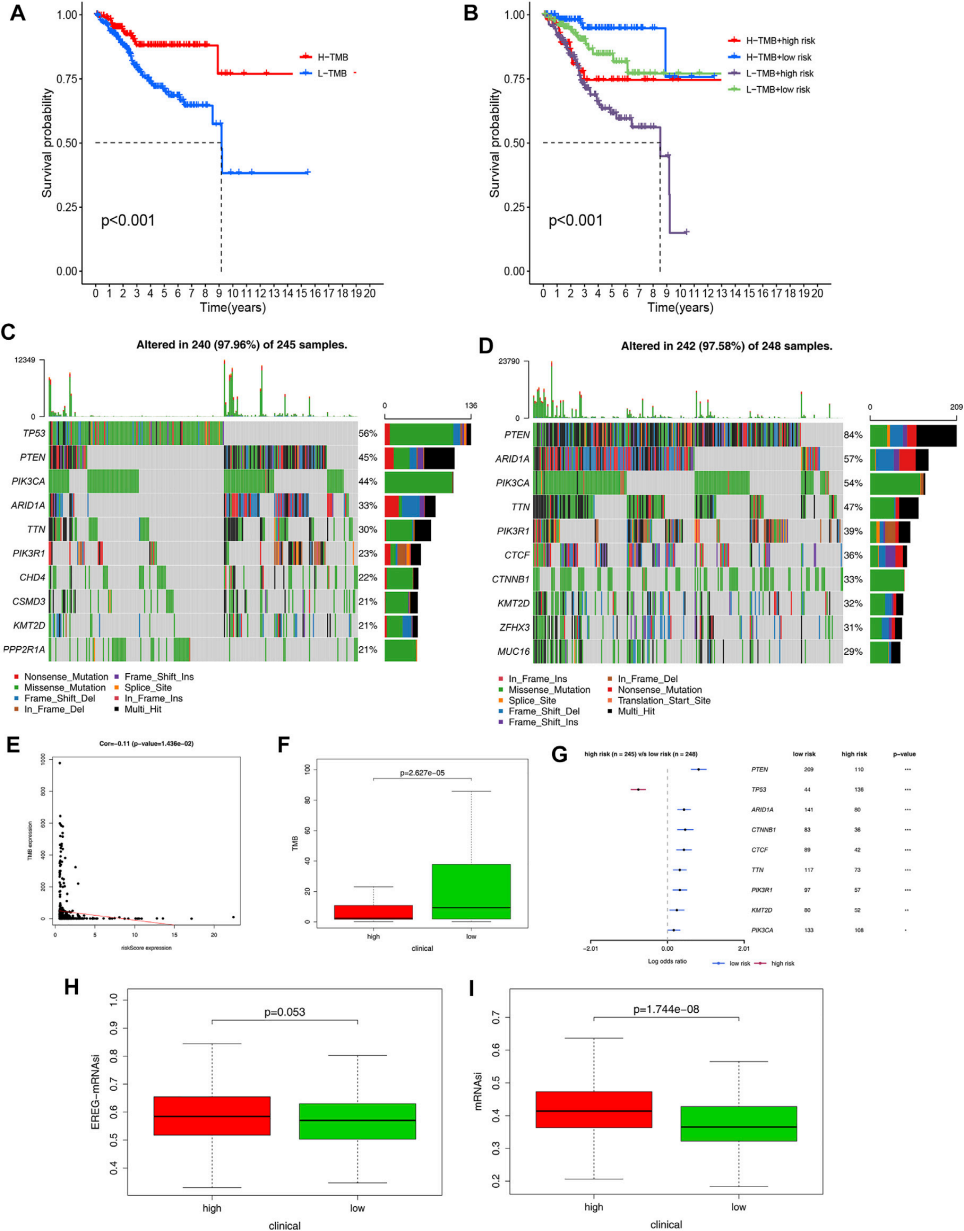
TMB level and Somatic mutation analysis. **(A)** Survival analysis between patients with high- and low-TMB EC. **(B)** Two-factor survival analyses of risk score and TMB levels. **(C,D)** Landscape of mutation profiles was shown in the waterfall plot, in which the type of mutation is shown in the comment bar (bottom) and genes are ordered by their mutation frequency. **(E)** Relationship between tumor mutation burden (TMB) and immune-related risk score. **(F)** Differences of tumor mutation burden in the high-risk and low-risk groups. **(G)** Differences of mutated genes between the high- and low-risk groups. **(H,I)** mRNA stemness indices between the high- and low-risk groups.

To investigate the prognostic value of TMB, we calculated the TMB value of EC samples in the TCGA database and divided them into high-TMB and low-TMB groups based on the median TMB value. The survival rate of the two groups was analyzed using the K-M curves and the results showed the best prognosis in patients with EC with high-TMB (Figure 8A). When the risk score and TMB analyses were combined, it was revealed that patients with EC with low-TMB and high-risk scores had the worst prognosis, whereas patients with EC with high-TMB and low-risk scores had the best prognosis (Figure 8B). Furthermore, we analyzed genome-wide mutations in EC high- and low-risk groups and found that missense mutations were the most common among different types of mutations, single-nucleotide polymorphisms were more common than deletions or insertions, and C > T transitions accounted for the highest proportion in both groups. Horizontal histogram demonstrated that the top 10 mutated genes in the two groups differed, but PTEN was among the top two (Supplementary Figure S6). In the high-risk group, 10 genes were mutated at a rate of more than 21%: TP53 (56%), PTEN (45%), PIK3CA (44%), ARID1A (33%), TTN (30%), PIK3R1 (23%), CHD4 (22%), CSMD3 (21%), KMT2D (21%), and PPP2R1A (21%). In the low-risk group, 10 genes were mutated at a rate of more than 29%: PTEN (84%), ARID1A (57%), PIK3CA (54%), TTN (47%), PIK3R1 (39%), CTCF (36%), CTNNB1 (33%), KMT2D (32%), ZFHX3 (31%), and MUC16 (29%) (Figures 8C,D). Moreover, we found that the risk score was negatively correlated with TMB (*p* = 0.01426, Figure 8E). TMB appeared to be higher in patients with EC in the low-risk group than in those in the high-risk group (Figure 8F). We also discovered that the low-risk group had more samples with PTEN, ARID1A, CTNNB1, CTCF, TTN, PIK3R1, KMT2D, and PIK3CA mutations than the high-risk group (Figure 8G). The mRNA stemness indices in the high-risk group were higher than those in the low-risk group, and there was no difference in the EREG-mRNA stemness indices between the two groups (Figures 8H,I).

### Prognostic lncRNAs Expression and Cancer Cell Sensitivity to Chemotherapy

**FIGURE 9:**
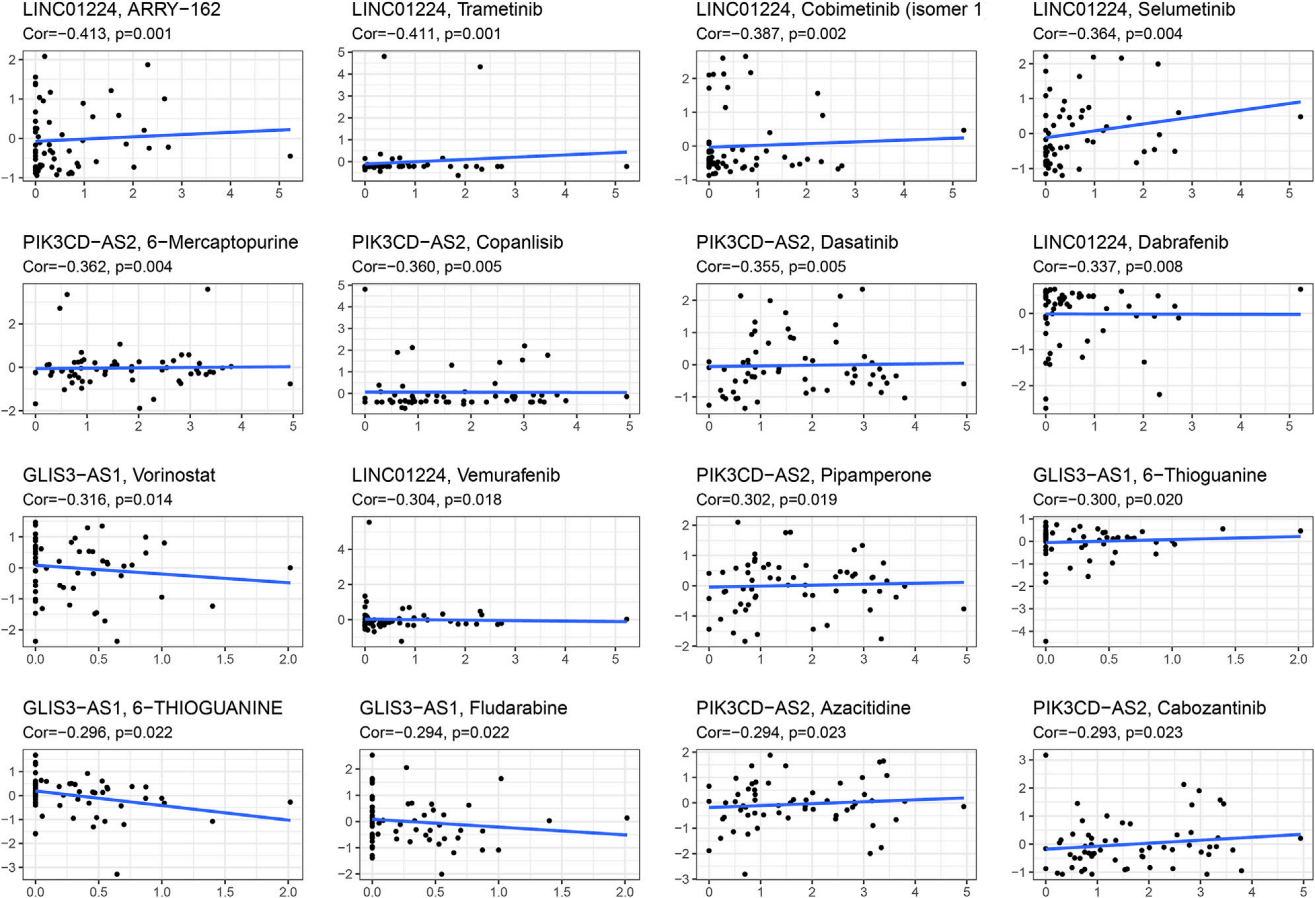
Scatter plot of relationship between the expressions of lncRNAs related to genetic instability and drug sensitivity.

The IC50 of each EC sample was calculated and compared using a common chemical drug prediction model. We screened 20 chemotherapeutic drugs with lower IC50 (including Bicaluramide, AKT.inhibitor.VIII, X17.AGG, RDEA119, Bryostatin.1, FTI.277, AZD6244, XMD8.85, PD.0325901, Roscovitine, SB.216763, CHIR.99021, AZD6482, PF.562271, PD.0332991, Embelin, Bexarotene, Nutlin.3a, BMS.708163, Lapatinib, FH535, AZ628, BMS.754807, BMS.536924, LFM.A13) in comparison to the high-risk group (*p* < 0.05) (Supplementary Figure S7). Furthermore, we investigated the correlation of expression of the lncRNAs related to genetic instability in NCI-60 cell lines and the relationship between expression and drug sensitivity. The analysis demonstrated that increased LINC01224 expression was associated with decreased drug sensitivity of cancer cells to ARRY-162, Trametinib, Cobimetinib, Selumetinib, Dabrafenib, and Vemurafenib, whereas increased PIK3CD-AS2 expression was associated with increased drug resistance of cancer cells to 6-Mercaptopurine, Copanlisib, Dasatinib, and Pipamperone. Furthermore, as GLIS3-AS1 expression increased, cancer cell drug sensitivity to Vorinostat and 6-Thioguanine decreased (Figure 9). The results showed that lncRNAs associated with genome instability were potentially related to chemotherapy drug sensitivity (*p* < 0.05).

### Validating Genome Expression Levels of Instability-Related lncRNAs in EC Samples

To validate the expression levels of genome instability-related lncRNAs, we used qRT-PCR to detect the expression levels of five genome instability-related lncRNAs in 15 EC samples and 15 normal tissues. The findings revealed that PIK3CD-AS2 was significantly downregulated, whereas AC129507.4 was highly expressed (Supplementary Figures S8A,B). However, there was no difference in the expression of GLIS3.AS1, LINC01224, and AC007389.3 between EC and normal samples (Supplementary Figures S8C–E).

## Discussion

Globally, EC is among the most common female genital cancers and the prevalence of EC continues to increase every year. The lack of significant improvement in the 5-year survival rate for patients with EC suggests that the disease remains a serious threat to female health (Bray et al., 2018; Feng et al., 2019) Therefore, there is an urgent need for better surveillance and treatment programs for EC. The current study was aimed at identifying the GILncSig and its prognostic value in EC.

To investigate the value of lncRNA-related genomic instability on the prognosis of patients with EC, the patients were grouped based on mutation frequency and filter the target genes. First, a total of 109 lncRNAs with different expression levels in the quarter highest and guarter lowest mutation frequency samples were identified. The lncRNAs were then screened to be associated with genomic instability in patients with EC. In addition, it was also investigated whether the genomic instability-associated lncRNAs could predict clinical outcomes. Results of the present study showed that patients in the high-risk group had the worse outcomes and lower somatic mutation, which was validated by the testing set as well as the TCGA set.

Results of the ROC curve analysis show that the clinical factor combined with risk score exhibited the most significant effect in the model as compared with other models. The construction and validation of GILncSig revealed that GILncSig effectively predicted the prognosis and gene instability of patients with EC. Further, among the numerous lncRNAs, lncRNAs AC007389.3, PIK3CD-AS2, LINC01224, AC129507.4, and GLIS3-AS1 were included in the prognosis model employed in the current study. It has been reported that LINC01224 is highly expressed in epithelial ovarian cancer and can promote the development of the cancer through miR-485-5p–mediated PAK4 (Gong et al., 2020). Therefore, interference with the expression of LINC01224 can inhibit the proliferation of colon cancer cells and induce cell cycle arrest in G0-G1 phase (Xing et al., 2020). The PIK3CD-AS2 lncRNAs also promotes tumor cell proliferation and inhibit apoptosis, and this is thus associated with poor prognosis of lung adenocarcinoma (Zheng et al., 2020). On the other hand, GLIS3-AS1 lncRNAs may be a biomarker for pancreatic duct adenocarcinoma (Permuth et al., 2017).

In addition, it was evident that the largest difference in mutations between these two groups of lncRNAs (PIK3CD-AS2 and GLIS3-AS1) was PTEN mutation, which was more common in the low-risk samples than in the high-risk samples. A previous study conducted by Erkanli et al. reported that the mutation or deletion rate of PTEN protein in Ⅰ type EC was between 34% and 55%, and even >80% (Erkanli et al., 2006). Moreover, the analysis of PTEN gene mutation in patients with EC and precancerous lesions revealed that 83% of the patients had PTEN mutation and 55% of precancerous lesions also had genetic mutation, whereas normal endometrial tissue samples did not have PTEN mutation (Mutter et al., 2000). The results of the present study indicated that low-risk patients with more PTEN mutation have worse prognosis outcome than high-risk patients with fewer PTEN mutations. Therefore, the enrichment of co-expressed genes was associated with carcinogenesis, and genome stability as well as immune and genomic instability-related lncRNAs were found to predict the prognosis and be an indicator of genomic instability of patients with the cancer.

Currently, with the increase in understanding of the tumor microenvironment, the importance of and the role that immune cells play has been extensively elucidated. A previous study on head and neck cancer (HNC) has confirmed that some immune cells, such as M0 macrophages and NK cells, have longer dormancy time in tumor tissues than in the adjacent normal tissues, whereas other cells, such as B cell naïve, are shorter and thus are significantly relevant in the HNC survival (Xie et al., 2018; Liu et al., 2020b). Unlike other tumor-associated macrophages, it has been reported that M0 macrophages play a specific role in survival of patients with HNC.

In pancreatic ductal adenocarcinoma, Cansu et al., through tumor proliferation and apoptosis assay of their study, showed that M0 macrophages harbor anti-tumorigenic activities that M1 and M2 did not share (Helm et al., 2014; Tekin et al., 2020). In addition, investigation carried out by Peipei et al. demonstrated that NK cells are regulated by lncRNA GAS5 through upregulation of the expression of miR-544 to perform its role as liver cancer killer (Fang et al., 2019). Elsewhere, Mohsen et al. reported that the number of memory B cells, which can express granzyme B (GZMB), was smaller in metastatic as compared with non-metastatic lymph nodes, and this hence emphasized the importance of GZMB + b cells in patients with breast invasive ductal carcinoma (Arabpour et al., 2019; Chesneau et al., 2020). Cytokine negatively regulates IFNγ signaling through CSF1R expression, thus modulating the functions of tumor infiltration (Wang et al., 2020b).

Immune-microenvironment of the tumor has been considered to be crucial in the progression of the tumor and response to therapies. The present study showed that patients in the low-risk group were more prone to mutations as compared with those in the high-risk group. Further analysis confirmed that the risk score was positively correlated with tumor immune-related cells and negatively associated with TMB and MSI. In addition, it has been found that the immune checkpoint inhibitors prevent tumors from activating immune checkpoint protein receptors on the surface of immune cells. This prevented tumors from evasive immune responses and cause of the immune system to produce an anti-tumor response (Intlekofer and Thompson, 2013). In addition, some studies have shown a breakthrough in the treatment of advanced EC with a combination of PD-L1 inhibitor Pembrozumab and Lenvatinib (Pakish and Jazaeri, 2017; Makker et al., 2019; Makker et al., 2020).

Results of the present study found that 25% of patients with EC were associated with defects of DNA MMR, which is characterized by an error in nucleotide repeats during DNA replication, also referred to as microsatellite instability (MSI) (Le et al., 2015). MMR defects led to a higher rate of somatic cell mutation, which increased the tumor antigen load and the number of tumor-infiltrating lymphocytes of these tumors with MMR defects, corresponding to the increased expression of PD-1 as well as PD-L1 (du Rusquec et al., 2019; Howitt et al., 2015). Therefore, the high incidence of MSI in EC has promoted application of immunosuppressive agents as a new therapeutic intervention. The validation results hence indicated that the GILncSig not only predicts the prognosis and genomic instability but also is an indicator of immune infiltration of patients with EC.

The relationship between the effectiveness of chemo-drug and genomic instability-related lncRNAs on different risk groups was predicted by GDSC database. Results of the present study showed that LINC01224 was negatively associated with reduced sensitivity of cancer cells to drug such as ARRY-162, Trametinib, Cobimetinib, Selumetinib, Dabrafenib, and Vemurafenib. Further, ARRY-162 and Trametinib, as well as Cobimetinib and Selumetinib are the reversible inhibitor of MEK1 and MEK2 (Lugowska et al., 2015). In addition, it was evident that Trametinib inhibits BRAF V600 mutation-positive melanoma cell growth both *in vitro* and *in vivo* through activating MEK1/2 to regulate ERK pathway, which had been approved by Food and Drug Administration (FDA) to treat BRAF V600E or V600K mutation-positive melanoma and non–small cell lung cancer (Geraud et al., 2020; Roskoski, 2020). Meanwhile, Dabrafenib and Vemurafenib are also effective drugs for the treatment of melanoma (Chapman et al., 2011; Hauschild et al., 2012).

The increased expression of PIK3CD-AS2 was associated with increased drug resistance of cancer cells to 6-Mercaptopurine, Copanlisib, Dasatinib, and Pipamperone. Furthermore, drug sensitivity of cancer cells to Vorinostat and 6-Thioguanine was downregulated as the expression of GLIS3-AS1 was increased. It was also suggested that 6-mercaptopurine can be used as a maintenance therapy for acute lymphoblastic leukemia (Bell et al., 2004; Escherich et al., 2011). Results of the present study found that Copanlisib is a generic I type PI3K inhibitor with significant activity against PI3Kα and PI3Kδ subtypes, both of which play important roles in B-cell malignancies (Decaudin et al., 1999; Swerdlow et al., 2016).

Follicular lymphoma (FL) is a common form of indolent non-Hodgkin’s lymphoma, and the FDA has accelerated approval of copanlisib for the treatment of relapsed or refractory FL in adults who have received at least two systemic treatments (Magagnoli et al., 2020). Therefore, the reported correlation between the genomic instability-related lncRNAs and chemotherapy drugs suggested that GILncSig exhibited high therapeutic potential in patients with EC. The results of the present study also demonstrated that genomic instability-related lncRNAs can be used as therapeutic targets to overcome drug resistance or adjuvant drug sensitivity.

However, the current study has some limitations. First, patients in the representative samples were predominantly Western people, and thus, so whether the research results need to be verified if they hold true for Asians and other people in the world. Second, the conclusions of the present study may exhibit deviation because some clinical indicators records were incomplete. Furthermore, the preliminary findings still require to be further validated on a larger cohort and with biological experiments.

## Conclusion

In conclusion, the present study identified and validated the GILncSig. Further, the prognostic prediction, somatic mutation, and chemotherapeutic drug sensitivity were performed to explore the roles of GILncSig in patients with EC. Therefore, the current study provided a critical approach of investigating the prognostic role of genome instability-related lncRNA, particularly in the areas of immune response, tumor microenvironment, and drug resistance, which is essential for the development of personalized cancer therapies.

## Data Availability

The datasets presented in this study can be found in online repositories. The names of the repository/repositories and accession number(s) can be found in the article/Supplementary Material.

## Supplementary Material

Supplementary Figure 1 Heatmap of 20 most significantly upregulated lncRNAs and 20 most significantly downregulated lncRNAs between GS and GU group.

Supplementary Figure 2 Relationship between the expressions of five genetic instability-related lncRNAs and clinicopathological features.

Supplementary Figure 3 Functional enrichment of transcript message of genomic instability-related lncRNAs in the high- and low-risk groups.

Supplementary Figure 4 Stratified analyses of clinicopathological factors in EC between the high-risk and low-risk groups: **(A,B)** age, **(C,D)** grade, and **(E,F)** histological type.

Supplementary Figure 5 Correlation between the expression of five prognostic lncRNAs and tumor-infiltrating immune cells (TICs).

Supplementary Figure 6 Distribution of variants based on variant classification, type, and SNV class. Bottom part (from left to right) indicates mutation load for each sample and the top 10 mutated genes in patients with endometrial cancer **(A,B)**.

Supplementary Figure 7 Prediction of response to common chemo-drugs between the high-risk and low-risk groups based on GDSC database. IC_50_, half-maximal inhibitory concentration.

Supplementary Figure 8 Expression level of PIK3CD-AS2, AC129507.4, GLIS3.AS1, LINC01224, and AC007389.3 in clinical samples.
