# Supplementary figures and images for: Comprehensive Analysis of the Prognostic Signature of Mutation-Derived Genome Instability-Related lncRNAs for Patients With Endometrial Cancer
Source: Front Cell Dev Biol. 2022 Apr 1;10:753957. doi: 10.3389/fcell.2022.753957 (PMC9012522; doi:10.3389/fcell.2022.753957)

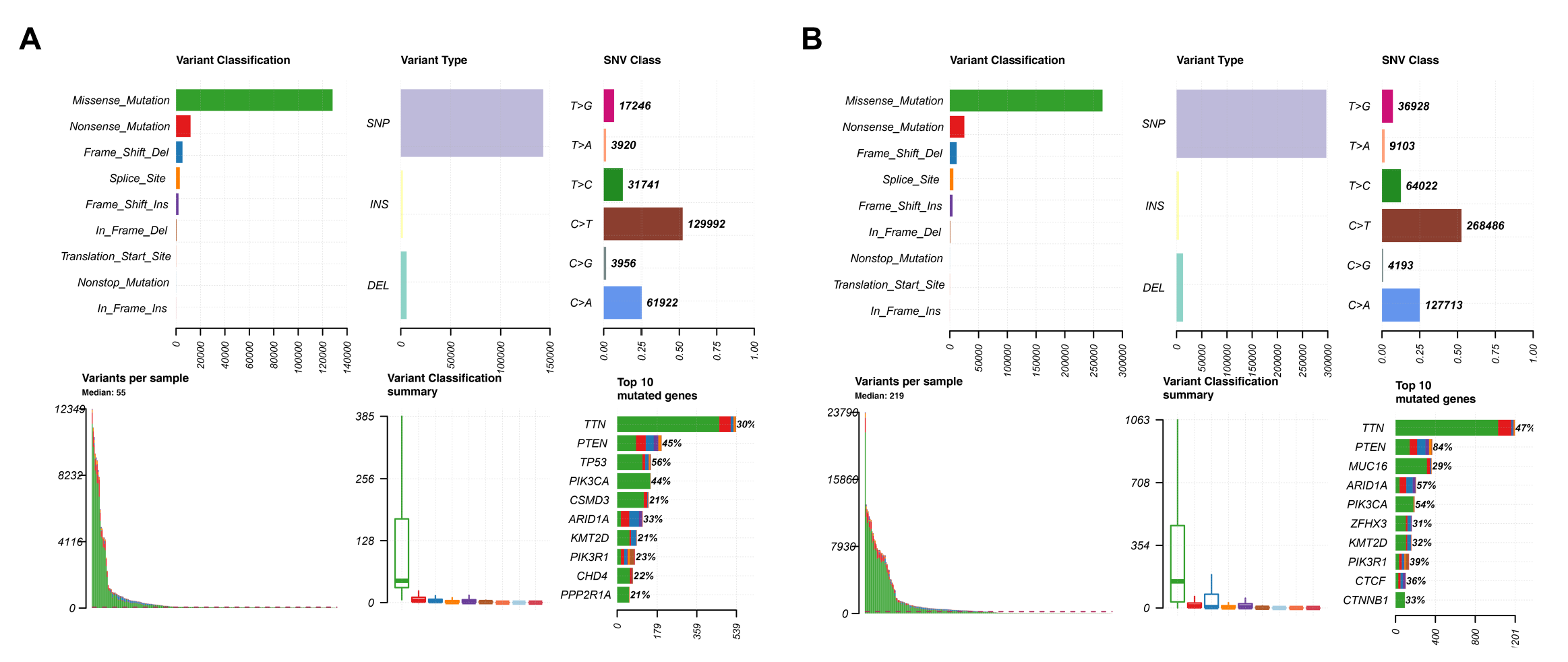

Supplement: Supplementary file 1 [file Image6.TIF]

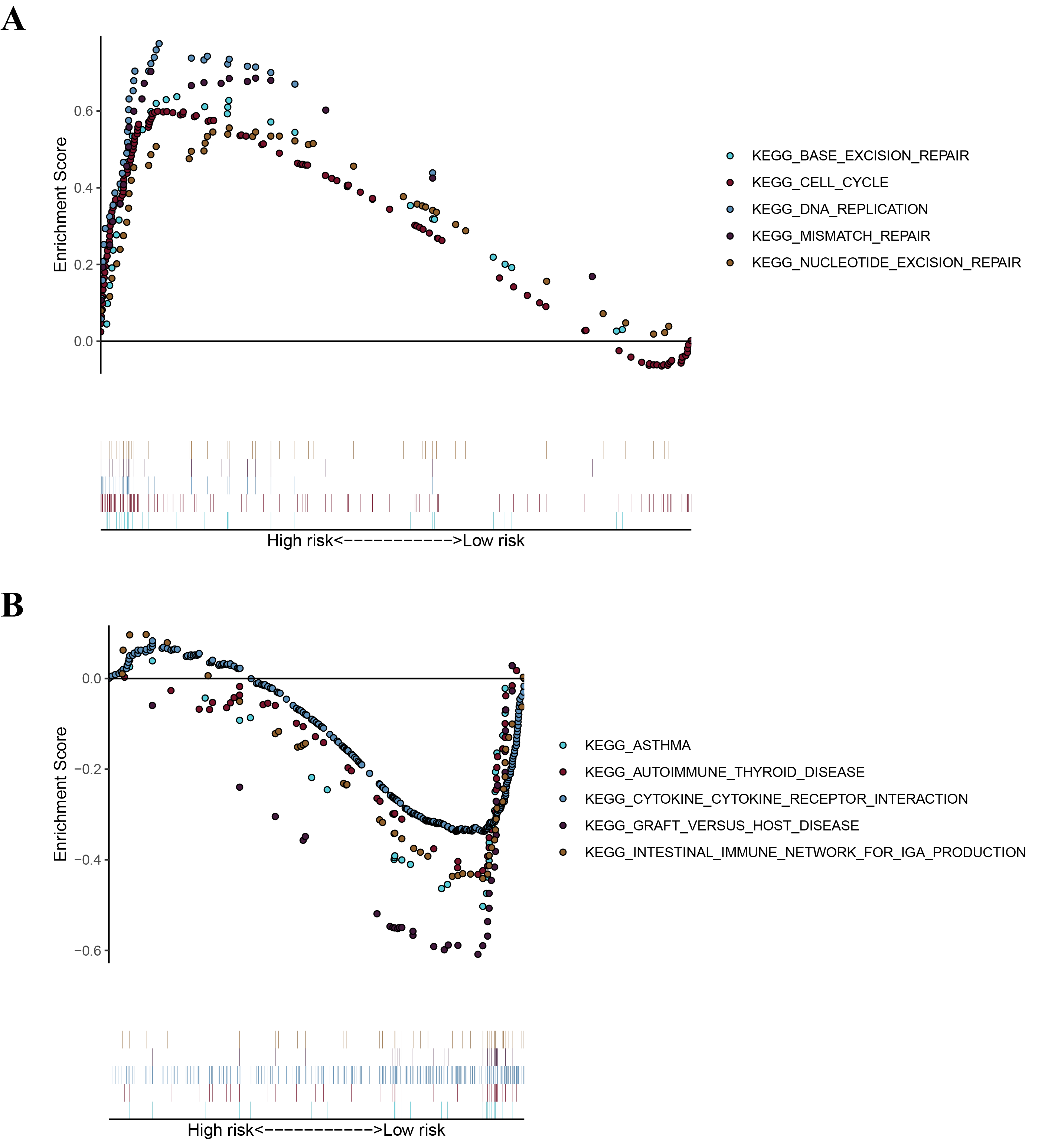

Supplement: Supplementary file 3 [file Image3.TIF]

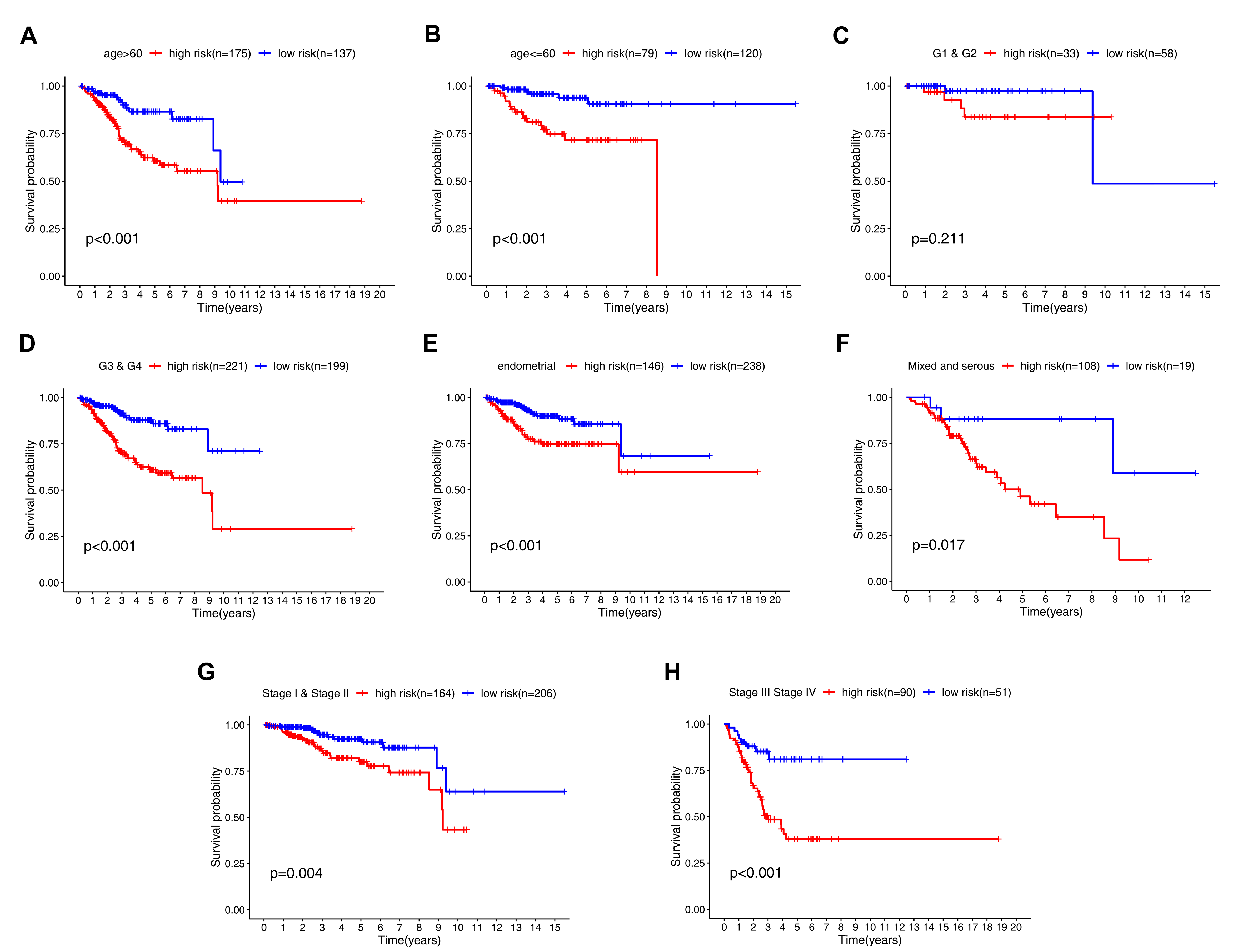

Supplement: Supplementary file 4 [file Image4.TIF]

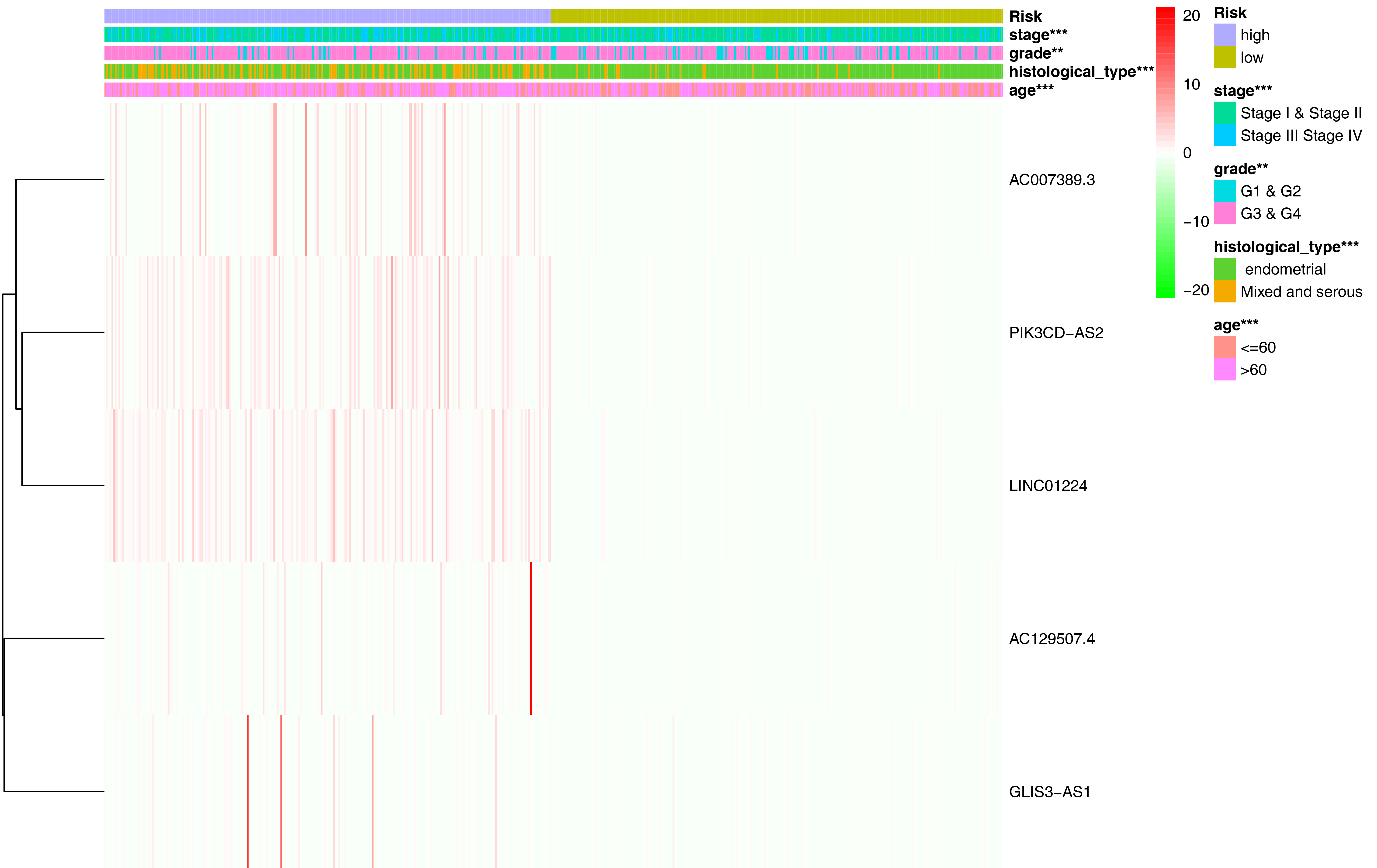

Supplement: Supplementary file 5 [file Image2.TIF]

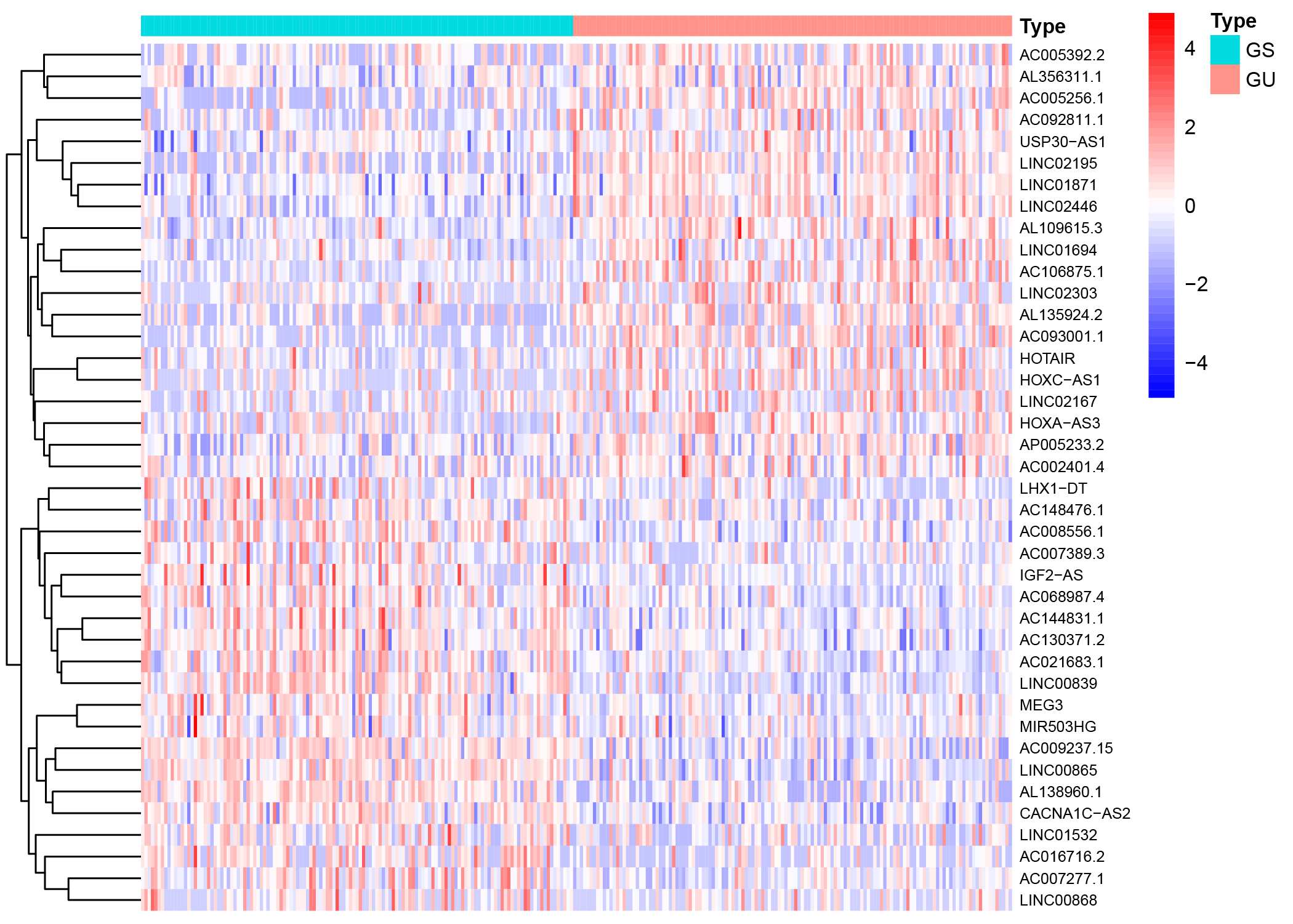

Supplement: Supplementary file 6 [file Image1.TIF]

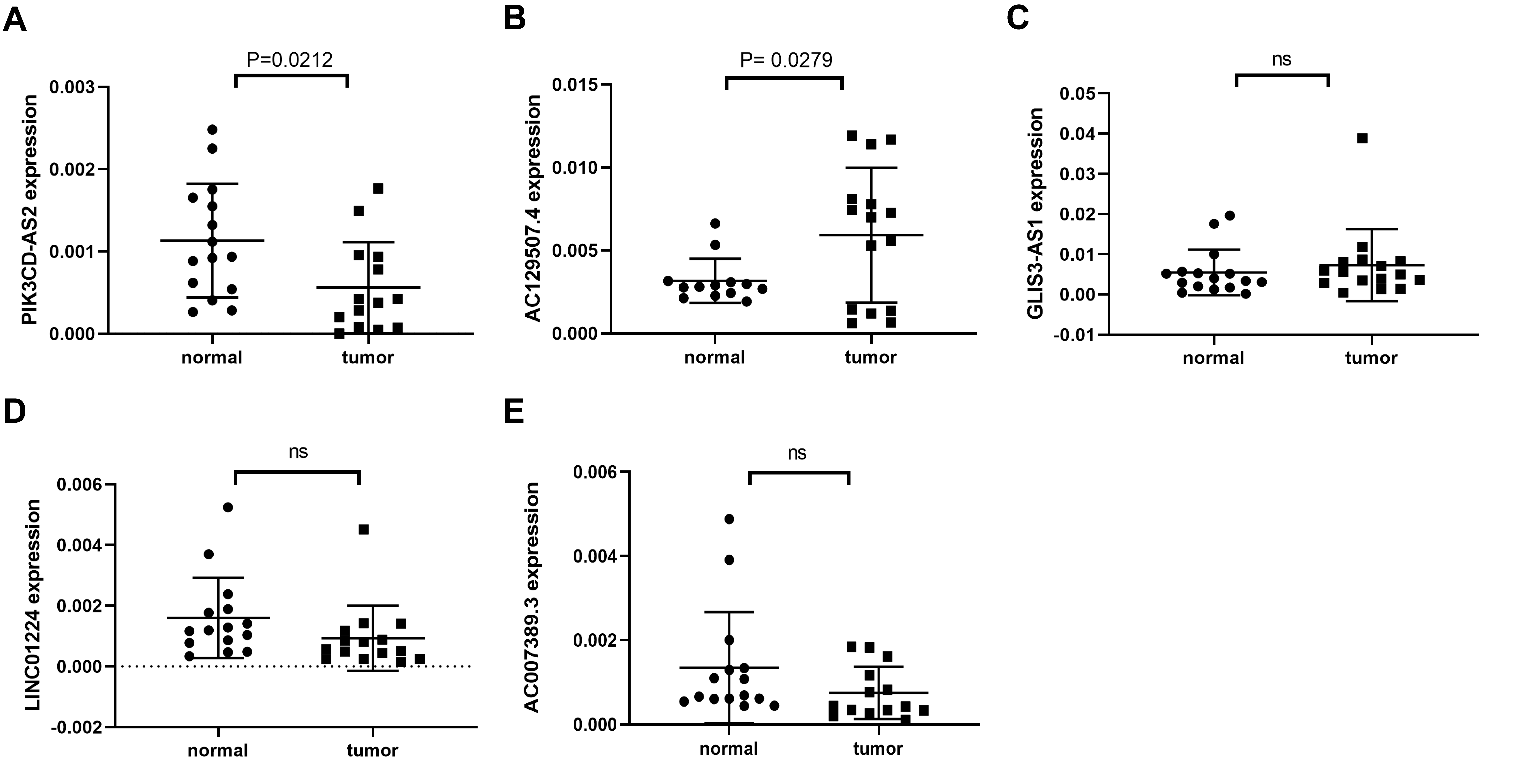

Supplement: Supplementary file 11 [file Image8.TIF]

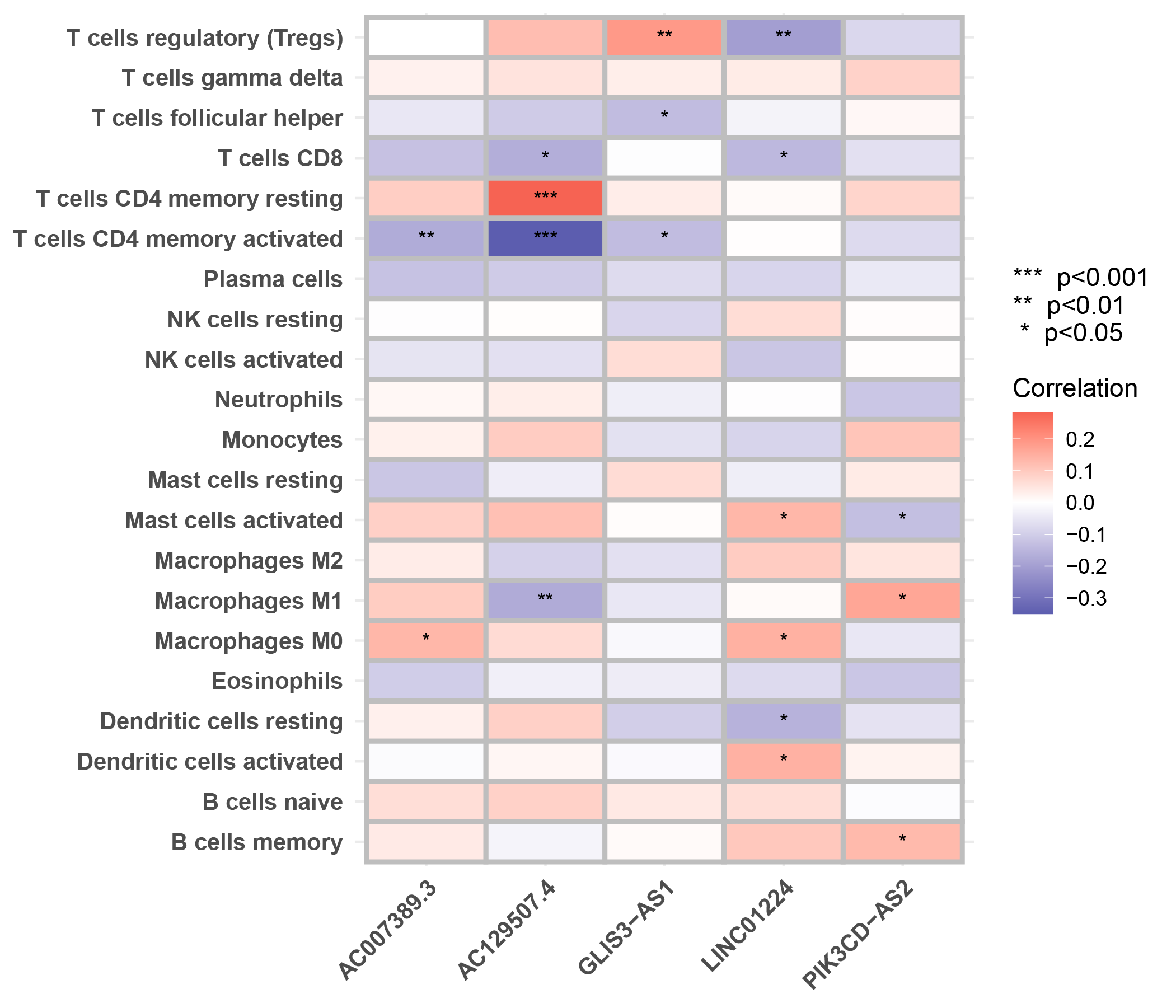

Supplement: Supplementary file 12 [file Image5.TIF]
