# Supplementary material for: Comprehensive Analysis of the Prognostic Signature of Mutation-Derived Genome Instability-Related lncRNAs for Patients With Endometrial Cancer
Source: Front Cell Dev Biol. 2022 Apr 1;10:753957. doi: 10.3389/fcell.2022.753957 (PMC9012522; doi:10.3389/fcell.2022.753957)
Supplement: Supplementary file 2 [file Table1.docx]

**Supplementary Table 1. Primer sequence of lncRNA in qRT-PCR**

|  | Forward sequence | Reverse sequence |
| --- | --- | --- |
| LINC01224 | AGAGCTTGGGATCGCTTTCTG | TTACTCAGGTGCCTTTCCCAC |
| PIK3CD-AS2 | GGGATCATAAATGCTTGCTGTT | CGTATTAGTTACTGGTTGCTGTT |
| AC129507.4 | GCAGATCAAACCCCGGAGC | TAGTTCTTCAGGGTGCAGTGG |
| GLIS3-AS1 | TGAGCAACAGGAGAACCACTA | GGGAGTTCACATGGCAGGATA |
| AC007389.3 | CTGCGCCATTCTATCCACCC | ATAAGGTGCAGCAGAGGGTTC |
