# Supplementary material for: Comprehensive Analysis of the Prognostic Signature of Mutation-Derived Genome Instability-Related lncRNAs for Patients With Endometrial Cancer
Source: Front Cell Dev Biol. 2022 Apr 1;10:753957. doi: 10.3389/fcell.2022.753957 (PMC9012522; doi:10.3389/fcell.2022.753957)
Supplement: Supplementary file 8 [file Table2.docx]

***Supplementary table 2.*** 109 differentially expressed lncRNAs between the GU and GS groups.

| LncRNA | conMean | treatMean | logFC | pValue | FDR |
| --- | --- | --- | --- | --- | --- |
| IGF2-AS | 0.928248 | 0.045229 | -4.3592 | 2.14E-06 | 2.81E-05 |
| AC016716.2 | 3.075705 | 0.260091 | -3.56383 | 2.41E-07 | 4.51E-06 |
| AC007277.1 | 1.554144 | 0.196485 | -2.98363 | 0.000211 | 0.001334 |
| AC144831.1 | 1.603196 | 0.227533 | -2.8168 | 2.04E-07 | 4.04E-06 |
| AC007389.3 | 0.862247 | 0.1458 | -2.56411 | 0.002528 | 0.009428 |
| LHX1-DT | 1.780094 | 0.326544 | -2.44661 | 9.45E-05 | 0.000684 |
| AC021683.1 | 1.421297 | 0.304965 | -2.22049 | 2.65E-09 | 1.39E-07 |
| AC130371.2 | 2.601052 | 0.577971 | -2.17003 | 1.97E-10 | 1.55E-08 |
| LINC00868 | 0.709161 | 0.172803 | -2.03698 | 0.006285 | 0.019368 |
| LINC00839 | 2.499681 | 0.661278 | -1.91842 | 5.98E-16 | 1.13E-12 |
| AC148476.1 | 0.7257 | 0.19975 | -1.86118 | 0.000284 | 0.001704 |
| AC008556.1 | 0.884058 | 0.253284 | -1.80338 | 2.20E-07 | 4.20E-06 |
| MEG3 | 1.439938 | 0.428614 | -1.74826 | 3.67E-05 | 0.000314 |
| AL138960.1 | 0.816582 | 0.250738 | -1.70342 | 1.33E-13 | 5.13E-11 |
| CACNA1C-AS2 | 1.082428 | 0.33837 | -1.6776 | 1.39E-09 | 8.20E-08 |
| AC009237.15 | 2.209789 | 0.696257 | -1.66622 | 1.61E-13 | 5.13E-11 |
| AC068987.4 | 0.958181 | 0.302531 | -1.66321 | 4.07E-08 | 1.28E-06 |
| MIR503HG | 1.790775 | 0.596732 | -1.58543 | 0.002031 | 0.008077 |
| LINC01532 | 0.610271 | 0.206215 | -1.5653 | 0.00048 | 0.002559 |
| LINC00865 | 1.462989 | 0.503291 | -1.53945 | 3.44E-15 | 2.17E-12 |
| AF131215.7 | 1.01756 | 0.361488 | -1.49309 | 1.73E-08 | 6.69E-07 |
| AC010255.1 | 0.698837 | 0.24942 | -1.48638 | 2.47E-06 | 3.14E-05 |
| LINC02600 | 1.872749 | 0.67541 | -1.47132 | 2.16E-13 | 5.83E-11 |
| Z97200.1 | 1.430842 | 0.550655 | -1.37764 | 0.005581 | 0.01757 |
| AF131215.5 | 0.881133 | 0.340759 | -1.37061 | 5.45E-07 | 9.11E-06 |
| AC002511.2 | 0.826795 | 0.319748 | -1.3706 | 1.71E-06 | 2.32E-05 |
| AL161618.1 | 0.691516 | 0.268153 | -1.36671 | 0.002666 | 0.009873 |
| ZNF295-AS1 | 5.014797 | 1.955863 | -1.35839 | 0.001444 | 0.006216 |
| AC107294.3 | 0.972292 | 0.389967 | -1.31804 | 3.73E-07 | 6.47E-06 |
| AL121820.2 | 4.52117 | 1.82797 | -1.30645 | 5.61E-13 | 1.33E-10 |
| AC100803.2 | 0.69503 | 0.283364 | -1.29442 | 1.35E-07 | 2.90E-06 |
| AL390198.1 | 2.367573 | 0.970861 | -1.28607 | 1.06E-06 | 1.52E-05 |
| LINC00284 | 1.431319 | 0.593381 | -1.27032 | 1.33E-07 | 2.90E-06 |
| LINC01006 | 1.964187 | 0.818828 | -1.2623 | 8.64E-12 | 1.05E-09 |
| AC106900.2 | 1.470447 | 0.613468 | -1.2612 | 1.83E-06 | 2.46E-05 |
| GLIS3-AS1 | 2.680017 | 1.140083 | -1.2331 | 0.004696 | 0.01519 |
| BX322234.1 | 0.706084 | 0.302749 | -1.22172 | 4.73E-08 | 1.44E-06 |
| AC129507.4 | 3.064532 | 1.314341 | -1.22133 | 0.000169 | 0.001122 |
| LINC02562 | 1.264976 | 0.545334 | -1.2139 | 2.73E-07 | 4.96E-06 |
| AL161785.1 | 2.608089 | 1.127923 | -1.20932 | 1.53E-07 | 3.21E-06 |
| LINC01224 | 1.981829 | 0.861316 | -1.20222 | 6.04E-07 | 9.85E-06 |
| AF131215.6 | 1.709696 | 0.746654 | -1.19523 | 4.79E-08 | 1.44E-06 |
| IRAIN | 1.108043 | 0.485324 | -1.19099 | 0.000407 | 0.002247 |
| AC023983.2 | 0.650595 | 0.286135 | -1.18507 | 6.53E-07 | 1.05E-05 |
| AL590560.1 | 3.148872 | 1.394778 | -1.1748 | 0.000113 | 0.000799 |
| AL590094.1 | 0.680505 | 0.303151 | -1.16657 | 3.01E-08 | 1.05E-06 |
| AC004540.2 | 4.196659 | 1.876823 | -1.16095 | 0.020518 | 0.047497 |
| AC022509.2 | 2.792864 | 1.249863 | -1.15998 | 1.11E-06 | 1.59E-05 |
| AL662791.1 | 0.845961 | 0.379373 | -1.15698 | 1.58E-06 | 2.16E-05 |
| LINC00987 | 0.765019 | 0.346538 | -1.14248 | 1.46E-11 | 1.57E-09 |
| AC079210.1 | 0.559364 | 0.253979 | -1.13908 | 0.000192 | 0.001231 |
| LINC00323 | 0.594401 | 0.271248 | -1.13182 | 3.68E-07 | 6.43E-06 |
| AC026336.3 | 12.44331 | 5.752536 | -1.1131 | 0.000177 | 0.001164 |
| LINC01480 | 12.61207 | 5.833183 | -1.11245 | 0.002859 | 0.010485 |
| PIK3CD-AS2 | 2.058392 | 0.958281 | -1.103 | 8.01E-08 | 2.05E-06 |
| AC009237.14 | 3.005593 | 1.403195 | -1.09893 | 8.89E-12 | 1.05E-09 |
| AC016747.1 | 1.309308 | 0.617353 | -1.08464 | 0.000177 | 0.001164 |
| AL161669.3 | 1.186336 | 0.560834 | -1.08087 | 6.93E-07 | 1.11E-05 |
| PCAT19 | 4.376675 | 2.06922 | -1.08075 | 1.53E-08 | 6.00E-07 |
| AC002091.1 | 0.764461 | 0.363112 | -1.07403 | 1.08E-09 | 7.25E-08 |
| AC011447.3 | 0.562209 | 0.267448 | -1.07185 | 0.003821 | 0.013123 |
| MIR205HG | 2.453526 | 1.168486 | -1.07022 | 0.001587 | 0.006618 |
| AL157392.4 | 1.009309 | 0.481432 | -1.06796 | 7.21E-06 | 7.61E-05 |
| AC079145.1 | 0.907438 | 0.433063 | -1.06722 | 1.29E-08 | 5.17E-07 |
| AP001094.1 | 0.716662 | 0.343024 | -1.06298 | 8.73E-05 | 0.000646 |
| AC027281.1 | 1.602706 | 0.767881 | -1.06156 | 3.28E-05 | 0.000283 |
| ZNF667-AS1 | 5.660325 | 2.718132 | -1.05827 | 8.68E-13 | 1.82E-10 |
| CASC15 | 0.615154 | 0.29709 | -1.05005 | 4.90E-09 | 2.26E-07 |
| AP000251.1 | 1.794958 | 0.868566 | -1.04724 | 7.83E-10 | 5.48E-08 |
| AL049539.1 | 0.645162 | 0.312201 | -1.04719 | 0.000965 | 0.004558 |
| AP002360.3 | 4.103923 | 1.98729 | -1.0462 | 7.02E-05 | 0.000553 |
| UMODL1-AS1 | 0.773988 | 0.37582 | -1.04227 | 0.000571 | 0.002955 |
| LINC00626 | 1.892383 | 0.920059 | -1.04041 | 0.015089 | 0.038209 |
| AL161729.3 | 1.397849 | 0.683617 | -1.03195 | 0.01061 | 0.029048 |
| AC107464.3 | 1.284577 | 0.628728 | -1.03078 | 2.12E-07 | 4.12E-06 |
| LINC02593 | 2.403646 | 1.186635 | -1.01835 | 3.38E-06 | 3.99E-05 |
| DLGAP1-AS2 | 3.796319 | 1.874626 | -1.018 | 6.65E-05 | 0.00053 |
| AC013264.1 | 8.288559 | 4.132315 | -1.00417 | 1.24E-05 | 0.000122 |
| AC110285.6 | 0.406454 | 0.84355 | 1.053382 | 7.10E-05 | 0.000556 |
| AC073842.1 | 0.421164 | 0.880958 | 1.064691 | 0.000275 | 0.001661 |
| AC127521.1 | 0.396525 | 0.842118 | 1.08661 | 5.59E-07 | 9.26E-06 |
| AC004080.4 | 0.573804 | 1.221754 | 1.090323 | 0.00849 | 0.024475 |
| LINC02489 | 0.752154 | 1.63755 | 1.122439 | 0.006534 | 0.019971 |
| AC084864.1 | 0.25173 | 0.562172 | 1.159135 | 7.13E-08 | 1.87E-06 |
| LINC01943 | 0.256993 | 0.576241 | 1.164943 | 1.08E-07 | 2.50E-06 |
| LINC01612 | 0.462951 | 1.046134 | 1.176136 | 0.00848 | 0.024475 |
| MNX1-AS1 | 0.725744 | 1.644083 | 1.17975 | 1.25E-09 | 7.96E-08 |
| AP003390.1 | 0.268418 | 0.608993 | 1.181945 | 2.70E-07 | 4.95E-06 |
| AL365181.3 | 1.550635 | 3.59766 | 1.2142 | 0.00165 | 0.006835 |
| LINC02303 | 0.336263 | 0.840478 | 1.321622 | 0.000417 | 0.00229 |
| AL109615.3 | 0.793467 | 2.02035 | 1.348363 | 8.74E-11 | 7.50E-09 |
| HOXA-AS3 | 0.358826 | 0.920367 | 1.358926 | 0.001574 | 0.006598 |
| USP30-AS1 | 0.694226 | 1.835166 | 1.402433 | 0.000593 | 0.003028 |
| HOTAIR | 0.288376 | 0.802981 | 1.477414 | 0.000129 | 0.000897 |
| AL356311.1 | 1.161714 | 3.290701 | 1.502139 | 7.91E-05 | 0.000595 |
| LINC01694 | 0.234054 | 0.670689 | 1.518801 | 1.45E-05 | 0.00014 |
| AP005233.2 | 0.644096 | 1.86702 | 1.535389 | 8.78E-07 | 1.30E-05 |
| AL135924.2 | 0.257915 | 0.856619 | 1.731757 | 5.03E-08 | 1.48E-06 |
| AC005392.2 | 0.593153 | 2.143563 | 1.853535 | 0.010592 | 0.02904 |
| LINC01871 | 1.464731 | 5.355817 | 1.870471 | 5.48E-11 | 5.17E-09 |
| LINC02195 | 0.23545 | 0.931165 | 1.983613 | 1.26E-12 | 2.38E-10 |
| HOXC-AS1 | 0.23654 | 1.058926 | 2.162449 | 1.23E-07 | 2.72E-06 |
| AC092811.1 | 0.147002 | 0.675928 | 2.201038 | 0.000149 | 0.001006 |
| AC005256.1 | 0.398539 | 1.988281 | 2.31873 | 1.93E-10 | 1.55E-08 |
| AC002401.4 | 0.289758 | 1.502003 | 2.373968 | 0.009298 | 0.026056 |
| LINC02446 | 0.237421 | 1.321501 | 2.476655 | 1.63E-13 | 5.13E-11 |
| LINC02167 | 3.06609 | 24.08288 | 2.973536 | 0.000908 | 0.004323 |
| AC093001.1 | 0.092504 | 1.171526 | 3.662724 | 2.14E-15 | 2.02E-12 |
| AC106875.1 | 0.508035 | 6.958606 | 3.775797 | 4.47E-09 | 2.17E-07 |
