# Supplementary material for: Comprehensive Analysis of the Prognostic Signature of Mutation-Derived Genome Instability-Related lncRNAs for Patients With Endometrial Cancer
Source: Front Cell Dev Biol. 2022 Apr 1;10:753957. doi: 10.3389/fcell.2022.753957 (PMC9012522; doi:10.3389/fcell.2022.753957)
Supplement: Supplementary file 9 [file Table3.docx]

**Supplementary Table 3. Univariate Cox proportional hazards regression analysis of 14 of 109 prognostic lncRNAs in training set.**

| ID | HR | HR.95L | HR.95H | pvalue |
| --- | --- | --- | --- | --- |
| AC107294.3 | 1.334 | 1.027 | 1.734 | 0.031 |
| AL049539.1 | 1.429 | 1.076 | 1.898 | 0.014 |
| AC129507.4 | 1.028 | 1.005 | 1.050 | 0.015 |
| GLIS3-AS1 | 1.015 | 1.002 | 1.028 | 0.027 |
| AC009237.14 | 1.202 | 1.085 | 1.332 | 0.000 |
| AL390198.1 | 1.094 | 1.001 | 1.194 | 0.047 |
| AC021683.1 | 1.137 | 1.025 | 1.261 | 0.015 |
| PIK3CD-AS2 | 1.170 | 1.038 | 1.319 | 0.010 |
| AP000251.1 | 1.155 | 1.035 | 1.289 | 0.010 |
| LINC01224 | 1.232 | 1.100 | 1.380 | 0.000 |
| AL590094.1 | 1.421 | 1.040 | 1.942 | 0.027 |
| AC009237.15 | 1.161 | 1.068 | 1.263 | 0.001 |
| LINC01006 | 1.180 | 1.058 | 1.316 | 0.003 |
| AC007389.3 | 1.296 | 1.111 | 1.513 | 0.001 |
