# Supplementary material for: Comprehensive Analysis of the Prognostic Signature of Mutation-Derived Genome Instability-Related lncRNAs for Patients With Endometrial Cancer
Source: Front Cell Dev Biol. 2022 Apr 1;10:753957. doi: 10.3389/fcell.2022.753957 (PMC9012522; doi:10.3389/fcell.2022.753957)
Supplement: Supplementary file 10 [file Table4.docx]

**Supplementary Table 4.** Multivariate Cox proportional hazards regression analysis of 5 of 14 prognostic lncRNAs in training set.

| ID | coef | HR | HR.95L | HR.95H | pvalue |
| --- | --- | --- | --- | --- | --- |
| AC129507.4 | 0.034 | 1.034 | 1.010 | 1.058 | 0.005 |
| GLIS3-AS1 | 0.018 | 1.019 | 1.005 | 1.032 | 0.007 |
| PIK3CD-AS2 | 0.119 | 1.127 | 0.984 | 1.290 | 0.085 |
| LINC01224 | 0.173 | 1.189 | 1.047 | 1.350 | 0.008 |
| AC007389.3 | 0.217 | 1.242 | 1.046 | 1.474 | 0.013 |
